# Unravelling and engineering an operon involved in the side-chain degradation of sterols in *Mycolicibacterium neoaurum* for the production of steroid synthons

**DOI:** 10.1186/s13068-023-02376-2

**Published:** 2023-08-02

**Authors:** Yun-Qiu Zhao, Yong-Jun Liu, Lu Song, Dingyan Yu, Kun Liu, Ke Liu, Bei Gao, Xin-Yi Tao, Liang-Bin Xiong, Feng-Qing Wang, Dong-Zhi Wei

**Affiliations:** 1 State Key Laboratory of Bioreactor Engineering, Newworld Institute of Biotechnology, East China University of Science and Technology, 130 Meilong Road, Shanghai, 200237 China; 2 Shanghai Key Laboratory of Molecular Imaging, Shanghai University of Medicine and Health Sciences, Shanghai, 201318 China

**Keywords:** *Mycolicibacteria*, HBC sub-pathway, *atf1*, HBC operon, C-22 steroid, 4-HBC

## Abstract

**Background:**

Harnessing engineered *Mycolicibacteria* to convert cheap phytosterols into valuable steroid synthons is a basic way in the industry for the production of steroid hormones. Thus, C-19 and C-22 steroids are the two main types of commercial synthons and the products of C17 side chain degradation of phytosterols. During the conversion process of sterols, C-19 and C-22 steroids are often produced together, although one may be the main product and the other a minor byproduct. This is a major drawback of the engineered *Mycolicibacteria* for industrial application, which could be attributed to the co-existence of androstene-4-ene-3,17-dione (AD) and 22-hydroxy-23,24-bisnorchol-4-ene-3-one (HBC) sub-pathways in the degradation of the sterol C17 side chain. Since the key mechanism underlying the HBC sub-pathway has not yet been clarified, the above shortcoming has not been resolved so far.

**Results:**

The key gene involved in the putative HBC sub-pathway was excavated from the genome of *M. neoaurum* by comparative genomic analysis. Interestingly, an aldolase- encoding gene, *atf1*, was identified to be responsible for the first reaction of the HBC sub-pathway, and it exists as a conserved operon along with a DUF35-type gene *chsH4*, a reductase gene *chsE6*, and a transcriptional regulation gene *kstR3* in the genome. Subsequently, *atf1* and *chsH4* were identified as the key genes involved in the HBC sub-pathway. Therefore, an updated strategy was proposed to develop engineered C-19 or C-22 steroid-producing strains by simultaneously modifying the AD and HBC sub-pathways. Taking the development of 4-HBC and 9-OHAD-producing strains as examples, the improved 4-HBC-producing strain achieved a 20.7 g/L production titer with a 92.5% molar yield and a 56.4% reduction in byproducts, and the improved 9-OHAD producing strain achieved a 19.87 g/L production titer with a 94.6% molar yield and a 43.7% reduction in byproduct production.

**Conclusions:**

The excellent performances of these strains demonstrated that the primary operon involved in the HBC sub-pathway improves the industrial strains in the conversion of phytosterols to steroid synthons.

**Supplementary Information:**

The online version contains supplementary material available at 10.1186/s13068-023-02376-2.

## Background

Sterols are a group of steroidal compounds, such as cholesterol, ergosterol, and phytosterols, widely found in eukaryotes, which have irreplaceable roles in maintaining cell membrane function [1, 2]. Moreover, sterols can be further converted to diverse steroidal hormones in animals and plants to regulate their growth and development [3]. In nature, some microorganisms can convert sterols into a series of steroidal compounds that can be used as commercial precursors to synthesize various steroid hormones in the industry [4, 5]. Among these, *Mycobacteria* is a commonly used genus to convert cheap phytosterols into steroid synthons, such as C-19 and C-22 steroids, because of its powerful catabolic capacity on sterols [6]. The C-19 steroids are a type of metabolites of sterols with completely degraded C17 side chain, and the common commercial products are androstene-4-ene-3,17-dione (AD), androstene-1,4-diene-3,17-dione (ADD), and 9-hydroxyandrost-4-ene-3,17-dione (9-OH-AD) [7, 8].

In contrast to C-19 steroids, C-22 steroids are the catabolites of sterols after incomplete degradation of the C17 side chain, which retains an isopropyl side chain at the C17 position, such as 3-oxo-23, 24-bisnorcholesta-4-diene-22-oate (4-BNC), 22-hydroxy-23, 24-bisnorchola-4-dien-3-one (4-BNA), 22-hydroxy-23, 24-bisnorchol-4-ene-3-one (4-HBC) [9, 10]. Currently, both C-19 and C-22 steroids have been widely used in the industry for the synthesis of sex hormones, adrenocortical hormones, and progestational hormones [11, 12]. This phenomenon benefited from the significant progress of modification technology of *Mycolicibacteria* by metabolic engineering along with the catabolic pathway of sterols [13]. Moreover, the complex catabolic pathway of sterols has some obvious deficiencies in the strains used in industry, which significantly affect their application performance [14, 15]. One of the most prominent problems is the generation of byproducts, especially the co-generation of the C-19 and C-22 steroids in the engineered C-19 or C-22 steroid-producing strains [5, 16]. A previous study demonstrated that the concomitant generation of C-19 and C-22 steroids is due to the coexistence of two metabolic branches in the C17 side chain degradation of sterols that have been designated as the AD sub-pathway responsible for the generation of C-19 steroids and the HBC sub-pathway closely related to the formation of some C-22 steroids, such as 4-HBC [17]. Compared to C-19 steroids, the molecular mechanism of the HBC sub-pathway is not yet clarified. This makes it difficult to modulate the correlation between the C-19 and C-22 sub-pathways in the development of C-19 or C-22 steroid-producing strains by metabolic engineering. This is the main reason that the C-22 byproducts cannot be eliminated in the C-19 steroid-producing strains and the C-19 by-products in C-22 steroid-producing strains in the industry, which significantly reduces the yield of the target products and increases the difficulty of downstream isolation and purification. Therefore, to further improve the application performances of the engineered C-19 and C-22 steroid-producing strains in the industry, it is necessary to understand the key molecular mechanism involved in the HBC sub-pathway. Also, elucidating the metabolic mechanism underlying the HBC sub-pathway is valuable in blocking the production pathway of byproducts and augmenting the metabolic flux of the target products [10, 18].

The catabolism of sterols is a sophisticated process in microorganisms [19, 20]. In 2007, a cholesterol degradation gene cluster (StCAT-GC) consisting of 81 genes was identified in *Mycobacterium tuberculosis* [21], which further elucidated the molecular mechanisms involved in the conversion of sterols to steroid synthons in *Mycolicibacteria* [22]. The genes in StCAT-GC have been classified into three categories: steroid nucleus degradation, C17 side chain degradation, and sterol transport [23, 24]. Hitherto, the molecular mechanisms of steroid nucleus degradation and sterol transport have been well identified [25, 26]. To develop the strains for the production of C-19 and C-22 steroids, some key genes, including those encoding the ketosteroid-Δ^1^-dehydrogenases homologs (KstDs) and/or 3-ketosteroid-9α-hydroxylases homologs (KshAs), involved in steroid nucleus degradation need to be blocked [27, 28]. Therefore, the degradation process of the C17 side chain of sterols is essential for the production of C-19 or C-22 metabolites [29, 30]. The degradation of the C17 side chain is similar to the β-oxidative degradation of fatty acids, and the conversion process from sterols to C-19 steroids is known as the AD sub-pathway [31, 32]. The key genes involved in the AD sub-pathway are mainly located in StCat-GC. For instance, an intracellular growth-related (*igr*) operon consisting of six genes has been identified to play key roles in the initial and final steps of β-oxidative degradation of C17 side chain of sterols [33, 34]. However, there is still some ambiguity regarding the degradation process of C17 side chain of sterols, especially the generation mechanism of C-22 steroids, such as the valuable steroid synthon 4-HBC. Interestingly, C-22 steroids, such as 3-oxo-pregn-4-ene-20-carboxylic acid (3-OPC) and 3-oxo-4,17-pregnadiene-20-carboxylic acid (3-OPDC), could be obtained by disrupting some genes in the *igr* operon [35, 36]. However, a correlation between these C-22 steroids and the main C-22 steroid end-product 4-HBC has not been clarified, which is the main difficulty in engineering the sterol degradation pathway for the production of valuable C-19 or C-22 steroids, such as AD, 9-OHAD, and 4-HBC.

**Fig. 1:**
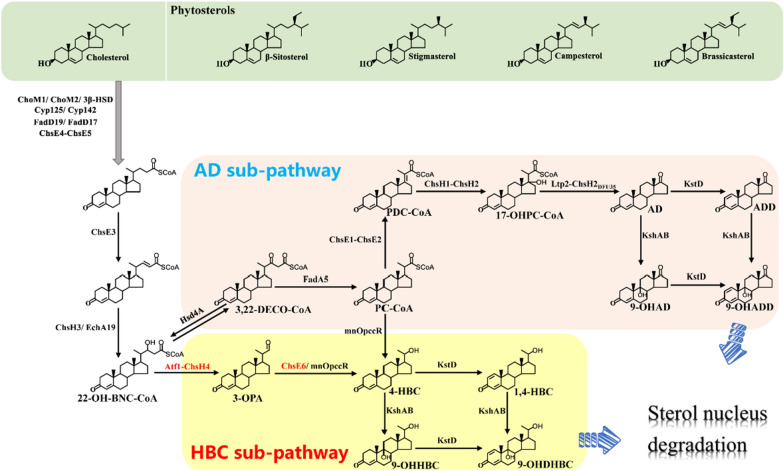
Proposed catabolic pathway of sterols in *M. neoaurum* ATCC 25795

To explore the generation mechanism of C-22 steroids, especially the valuable steroid synthon 4-HBC, we screened the putative genes related to the C17 side chain degradation of sterols in the gene cluster StCat-GC one by one in a previous study [18]. The results revealed that the knockdown of *hsd4A* gene resulted in a substantial decrease in the production of C-19 steroids and a significant increase in the formation of HBC-like products. Further investigation demonstrated that Hsd4A is a bifunctional enzyme, exhibiting the activity of 17β-hydroxysteroid dehydrogenase and 3β-hydroxy-acyl-coA dehydrogenase, and the deletion of *hsd4A* in an AD-producing strain could achieve a 4-HBC-producing strain [18]. These data suggested that *hsd4A* is a pivotal gene involved in the production pathway of C-19 steroids, catalyzing the conversion of 22-hydroxy-3-oxo-cholest-4-ene-24-carboxyl-CoA (22-OH-BNC-CoA) to 22–3-oxo-cholest-4-ene-24-carboxyl-CoA (22-oxo-BNC-CoA) in the consensus C17 side chain degradation pathway of sterols proposed previously [18]. This phenomenon determines the formation of the C-19 and the HBC-like C-22 steroids [37]. Therefore, the AD sub-pathway responsible for producing C-19 steroids and the HBC sub-pathway for producing C-22 steroids was proposed using 22-OH-BNC-CoA, the catalysis substrate of Hsd4A, as the branch point. This pathway was identified according to the characterized function of *hsd4A* and the changes in the chemical structure of these metabolites from 22-OH-BNC-CoA to C-19 and C-22 steroids (Fig. 1). Hitherto, the AD sub-pathway has been intensively investigated, and most of the involved key genes, such as *hsd4A*, *fadA5*, and the *igr* operon, are located in the well-characterized gene cluster StCat-GC [38, 39]. However, the proposed HBC sub-pathway with a putative conversion route from 22-OH-BNC-CoA to HBC-like steroids has not been confirmed, and the key genes involved in this pathway have yet to be identified.

Recently, a bifunctional reductase mnOpccR was identified with respect to the reduction in 3-oxo-4-pregnene-20-carboxyl-CoA (3-OPC-CoA) and 3-oxo-4-pregnene-20-carboxyl aldehyde (3-OPA) to 4-HBC in *Mycolicibacterium* CCTCC AB2019054 [40]. This finding provided an alternative mechanism for the production of HBC-like steroids. Nevertheless, this study also suggested the presence of the HBC sub-pathway. To confirm the putative HBC sub-pathway, the specific mechanism underlying the conversion of 22-OH-BNC-CoA into HBC-like steroids was investigated in this study. According to the reaction type and genomic and transcriptomic analyses, an aldolase gene *atf1* was identified to be involved in converting 22-OH-BNC-CoA to 3-OPCA, the first key reaction in the proposed HBC sub-pathway deduced from the genome of *M. neoaurum*. Further analysis revealed that *atf1* is distal from StCat-GC in the genome and shares the promoter with a DUF35-type scaffolding protein-encoding gene *chsH4*, a reductase gene *chsE6*, and a negative transcription factor *kstR3* as a typical operon. Subsequently, all the genes in the operon were identified as the key elements in HBC sub-pathway. Thus, this study confirmed that the putative HBC sub-pathway is real, and the revelation of the key genes involved in this pathway successfully provides a new and effective way to engineer strains in the conversion of phytosterols to C-19 or C-22 steroid synthons for industrial application. Therefore, an updated strategy was proposed to develop the engineered C-19 or C-22 steroid-producing stains by modifying the AD and HBC sub-pathways simultaneously. Finally, two improved strains for the production of 4-HBC and 9-OHAD were developed with a significantly enhanced production titer and molar yield and a substantially reduced proportion of byproducts.

## Results and discussion

### Identification of the key aldol lyase acting on the C22-23 cleavage in the HBC sub-pathway

4-HBC is a valuable product obtained from the catabolic process of sterols through the HBC sub-pathway in some species of *Mycolicibacteria* [18]. However, the key enzymes involved in the conversion of the HBC sub-pathway have not yet been identified. 22-OH-BNC-CoA has been identified as a crucial branching-point of the AD sub-pathway and the HBC sub-pathway in the catabolic process of sterols in our previous study (Fig. 1). According to the structural conversion of 22-OH-BNC-CoA to 4-HBC, the cleavage of 22-OH-BNC-CoA at C22-23 is considered an essential reaction to initiate the HBC sub-pathway and was presumed to be a retro-aldol reaction [18]. Thus, the enzyme involved in the reaction was identified from the genome of *M. neoaurum* ATCC 25795 (*Mn*), a powerful digestor of sterols. The analysis data revealed that the genes related to sterol catabolism were classified as the lipid transport and metabolism group, among which the enzymes that may catalyze the retro-aldol cleavage reaction were annotated as acetyl-CoA acetyltransferases (Atf). Then, 18 candidate *atf* genes were screened from 294 genes of the lipid transport and metabolism group in the genome of *Mn*.

**Fig. 2:**
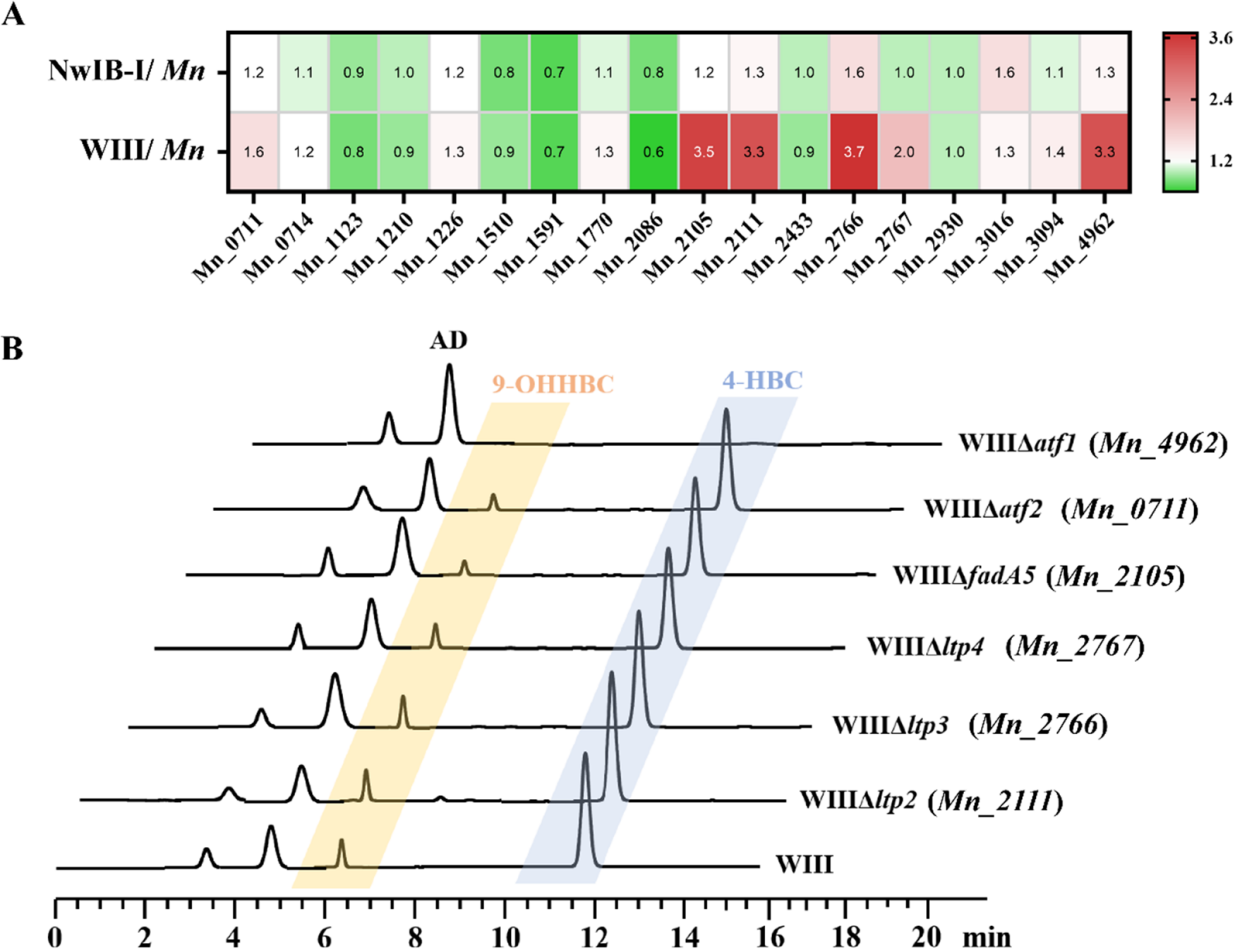
Identification of the key aldol lyase gene involved in the C22-23 cleavage in the HBC sub-pathway. **A** Expression heat-maps of 18 candidate aldol lyase genes in strains NwIB-I and WIII against strain *Mn*. **B** HPLC profiles of the strains with different aldol lyase genes knockout in the conversion of phytosterols to 4-HBC

To reduce the number of *atf* candidates, the expression differences of these candidates in the conversion process of sterols were compared among strains *Mn*, NwIB-I (9-OHAD-producing strain), and WIII (4-HBC-producing strain) based on the iTRIQ quantitative proteomics data (Fig. 2A). Then, 10 significantly upregulated *atf* genes in the WIII strain against *Mn* were filtered out. Of these, four genes did not show any significant differences in the expression between strains NwIB-I and WIII, suggesting that they might not have a clear correlation with the HBC sub-pathway. Finally, a candidate gene set composed of six *atf*s was obtained, including *Mn_2105*, *Mn_2111*, *Mn_2766* and *Mn_2767*, *Mn_0711*, and *Mn_4962*, among which *Mn_2105*, *Mn_2111*, *Mn_2766* and *Mn_2767* are located in the gene cluster of sterol degradation and labelled as *Mt_fadA5*, *Mt_ltp2*, *Mt_ltp3* and *Mt_ltp4*, respectively, while the other two genes, *Mn_0711* (designated as *atf2*) and *Mn_4962* (designated as *atf1*), have not been shown to be involved in the catabolism of sterols.

**Fig. 3:**
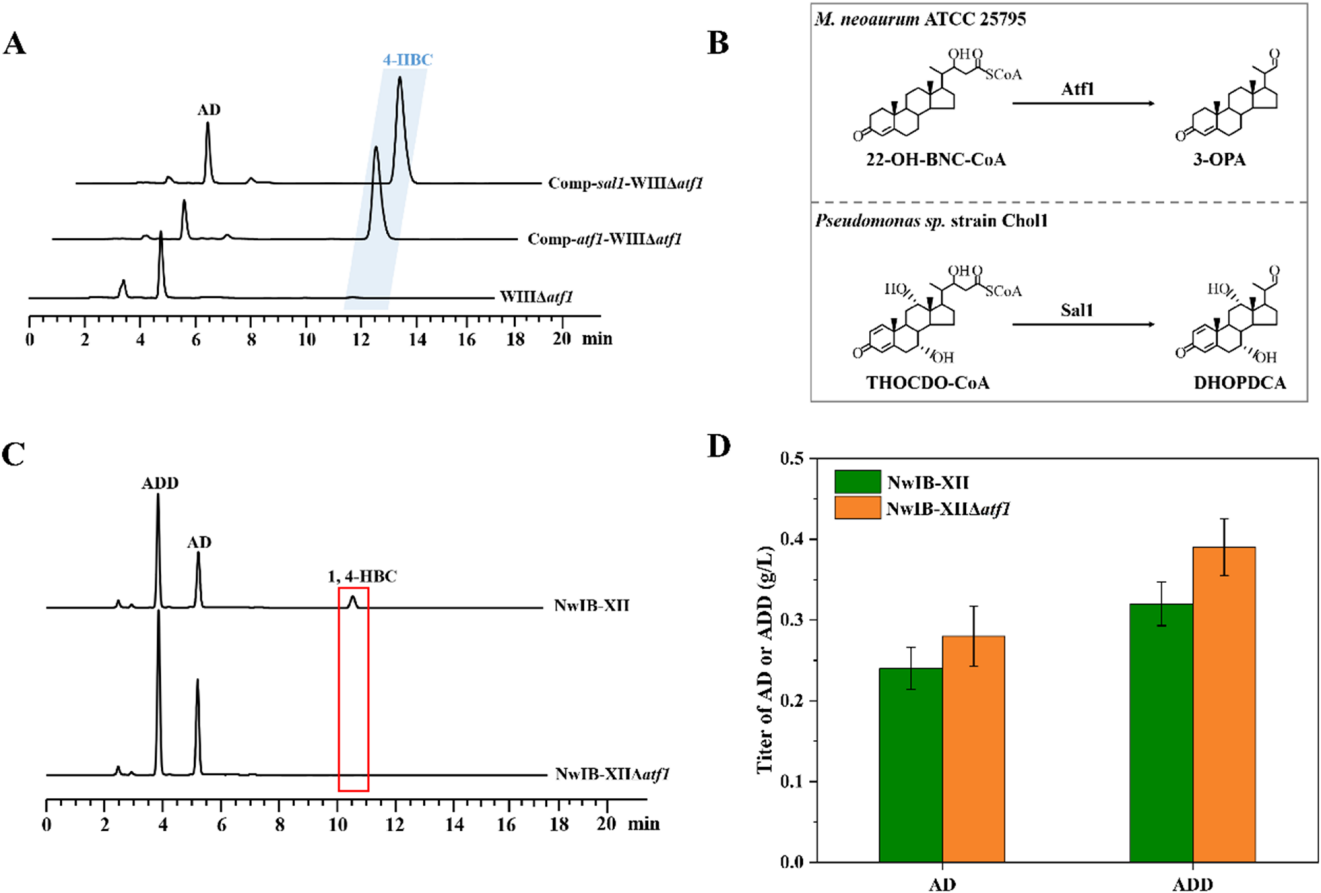
Role of Atf1 in the conversion of phytosterols to 4-HBC. **A** HPLC profiles of *atf1*-knockout strain (WIIIΔ*atf1*) and its functional complements (Comp-*atf1*-WIIIΔ*atf1* and Comp-*sal1*-WIIIΔ*atf1*) in the conversion of phytosterols to 4-HBC. **B** Schematic of the catalytic functions of Atf1 and Sal1. **C** HPLC profiles of strains NwIB-XIIΔ*atf1* and NwIB-XII in the conversion of phytosterols. **D** Effects of *atf1* deletion during the conversion of phytosterols to C-19 steroids (AD and ADD) in strain NwIB-XII. All assays were performed in triplicate with three independent measurements. Standard deviations of the biological replicates are represented by error bars

Subsequently, the potential roles of the six *atf*s in the HBC sub-pathway were evaluated by knockouts in strain WIII, generating strains WIIIΔ*fadA5*, WIIIΔ*ltp2*, WIIIΔ*ltp3*, WIIIΔ*ltp4*, WIIIΔ*atf1* and WIIIΔ*atf2*, respectively. The results showed that only strain WIIIΔ*atf1* failed to transform phytosterols to 4-HBC (Fig. 2B), indicating that the gene *atf1* might be associated with the HBC sub-pathway because the knockout of *atf1* completely blocked the generation of 4-HBC from the conversion of phytosterols. Then, the correlation between *atf1* and the HBC sub-pathway was confirmed by complementing *atf1* in strain WIIIΔ*atf1* (strain Comp-*atf1*-WIIIΔ*atf1*, Fig. 3A). These data clearly demonstrated that *atf1*, a putative gene encoding acetyl-CoA acetyltransferase, is a key molecule in the HBC sub-pathway.

To determine whether *atf1* plays a vital role in the retro-aldol cleavage of 22-OH-BNC-CoA at C22-23 in the HBC sub-pathway, the function of *atf1* was elucidated. Phylogenetic analyses showed that *atf1* is on a different branch of the phylogenetic tree from the other *atf1* candidates and has a close genetic association with *sal1* gene from *Pseudomonas* sp. Chol1 (Additional file 1: Figure S1A). The amino acid sequence alignment displayed 56% identity between Atf1 and Sal1 encoded by *atf1* and *sal1*, respectively, and both contained a conserved structural domain SCP-x_thiolase (Additional file 1: Figure S1B). The function of *sal1* has been characterized as an NAD^+^-dependent aldolase that plays a key role in the retro-aldol cleavage of 7α,12α,22-trihydroxy-3-oxochola-1,4-diene-24-acyl-CoA (THOCDO-CoA) at C22-23 in the catabolism of bile acid. Due to the structural similarity between C17 side chain of THOCDO-CoA and 22-OH-BNC-CoA (Fig. 3B), Atf1 might have a catalytic function similar to Sal1, which was consistent with our presumption of the function of Atf1 described above.

To substantiate the function of *atf1*, *sal1* from *Pseudomonas* sp. Chol1 was synthesized and introduced into strain WIIIΔ*atf1* as a substitute for *atf1* to determine whether the conversion phenotype of phytosterols to 4-HBC could be restored. The restored phenotype of the generated strain Comp-*sal*-WIIIΔ*atf1* indicated that *atf1* and *sal1* are identical in catalytic function, i.e., (Fig. 3A), similar to the physiological role of Sal1 on the retro-aldol cleavage of THOCDO-CoA, Atf1 effectuates the retro-aldol cleavage of 22-OH-BNC-CoA to produce 3-OPA (Fig. 3B).

Since 22-OH-BNC-CoA is the branching point of AD and HBC sub-pathways, it is also catalyzed by the key gene *hsd4A* in the AD sub-pathway besides *atf1* of the HBC sub-pathway (Fig. 1). Therefore, *hsd4A* and *atf1* might compete during the catabolism of sterols. To verify this phenomenon, *atf1* was deleted in the strain NwIB-XII, an engineered *Mn* with the production phenotype of AD, ADD, and 1,4-HBC during the conversion of sterols, to generate the strain NwIB-XIIΔ*atf1* (Table 1). Compared to the performance of NwIB-XII, the production of 1,4-HBC was blocked, which increased the yield of AD and ADD by 25% and 13% in the conversion of phytosterols by strain NwIB-XIIΔ*atf1* (Fig. 3C, D). These elevated yields could be attributed to the deletion of *atf1*, which blocks the metabolic flux of the HBC sub-pathway and shifts it to the AD sub-pathway (Fig. 1).

**Table 1:** Strains and plasmids used in this work

Name	Description	Source
*E. coli* DH5α	General cloning host	Transgen Biotech
*M. neoaurum* ATCC 25795	*M. neoaurum* type strain	ATCC
WIII	Deletion of *kshA1*, *hsd4A*, *kstD1*, *kstD2*, and *kstD3* in *M. neoaurum* ATCC 25795	[18]
NwIB-I	Deletion of *kstD1*, *kstD2*, and *kstD3* in *M. neoaurum* ATCC 25795	[13]
NwIB-XII	Deletion of *kshA1* and *kshA2* in *M. neoaurum* ATCC 25795	[18]
WIIIΔ*fadA5*	Deletion of *fadA5* in WIII	This work
WIIIΔ*ltp2*	Deletion of *ltp2* in WIII	This work
WIIIΔ*ltp3*	Deletion of *ltp3* in WIII	This work
WIIIΔ*ltp4*	Deletion of *ltp4* in WIII	This work
WIIIΔ*atf1*	Deletion of *atf1* in WIII	This work
WIIIΔ*atf2*	Deletion of *atf2* in WIII	This work
NwIB-XIIΔ*atf1*	Deletion of *atf1* deleted in NwIB-XII	This work
WIIIΔ*atf1*Δ*chsH4*	Deletions of *atf1* and *chsH4* in WIII	This work
WIIIΔ*kstR3*	Deletion of *kstR3* in WIII	This work
WIIIΔ*chsH4*	Deletion of *chsH4* in WIII	This work
WIIIΔ*chsE6*	Deletion of *chsE6* in WIII	This work
WIIIΔ*opccR*	Deletion of *opccR* in WIII	This work
WIIIΔ*chsE6*Δ*opccR*	Deletions of *chsE6* and *opccR* in WIII	This work
Comp-*atf1-*WIIIΔ*atf1*	*atf1* complemented in WIIIΔ*atf1*	This work
Comp-*sal1*-WIIIΔ*atf1*	*sal1* complemented in WIIIΔ*atf1*	This work
Comp-*chsH4-*WIIIΔ*chsH4*	*chsH4* complemented in WIIIΔ*chsH4*	This work
Comp-*shy-*WIIIΔ*chsH4*	*shy* complemented in WIIIΔ*chsH4*	This work
Comp-*sal1*-*shy-*WIIIΔ*chsH4*	*sal1* and *shy* complemented in WIIIΔ*chsH4*	This work
Comp-*sal1-*WIIIΔ*chsH4*	*sal1* complemented in WIIIΔ*chsH4*	This work
Comp-*atf1-*WIIIΔ*chsH4*	*atf1* complemented in WIIIΔ*chsH4*	This work
WIII-261-*kstR3*	*kstR3* over-expressed strain of WIII	This work
WIIIΔ*CSND*	Deletion of *CSND* gene cluster in WIII	This work
WIIIΔ*CSND*Δ*fadA5*	Deletion of *fadA5* and *CSND* gene cluster in WIII	This work
WIIIΔ*CSND*Δ*fadA5*-I	*atf1* over-expressed strain of WIIIΔ*CSND*Δ*fadA5*	This work
WIIIΔ*CSND*Δ*fadA5*-II	*chsH4* over-expressed strain of WIIIΔ*CSND*Δ*fadA5*	This work
WIIIΔ*CSND*Δ*fadA5*-III	*chsE6* over-expressed strain of WIIIΔ*CSND*Δ*fadA5*	This work
WIIIΔ*CSND*Δ*fadA5*-IV	*atf1* and *chsH4* co-overexpressed strain of WIIIΔ*CSND*Δ*fadA5*	This work
NwIB-IΔ*CSND*	Deletion of *CSND* gene cluster in NwIB-I	This work
NwIB-IΔ*CSND*-I	*kstR3* over-expressed strain of NwIB-IΔ*CSND*	This work
NwIB-IΔ*CSND*-II	Deletion of *atf1* in NwIB-IΔ*CSND*	This work
NwIB-IΔ*CSND*-III	Deletion of *chsH4* in NwIB-IΔ*CSND*	This work
NwIB-IΔ*CSND*-IV	Deletion of *chsE6* in NwIB-IΔ*CSND*	This work
NwIB-IΔ*CSND*-V	Over-expression of *kstR3* and deletion of *atf1* and *chsH4* in NwIB-IΔ*CSND*	This work

This result further confirmed the physiological function of *atf1* in the catabolic process of sterols that triggers the HBC sub-pathway. Thus, it can be deduced that the functional identification of *atf1* is significant for the development of optimized C-19 or C-22 steroid-producing strains through combined regulation of the metabolic fluxes of AD and HBC sub-pathways by inactivating or augmenting *atf1* and *hsd4A*.

### Identification of the HBC operon

Typically, the genes involved in the catabolism of steroids are usually found as gene clusters in organisms, such as the degradation clusters of sterols in *Mycolicibacteria* and *Rhodococcus* and the degradation cluster of cholate in *Pseudomonas* sp. Chol1 [41–43]. In the cholic acid degradation cluster, the aldolase *sal1* has been deciphered to be linked end-to-head with hydratase *shy*, forming a conserved operon among some steroid-degrading microorganisms and catalyzing a continuous two-step reaction in the C17 side chain degradation of cholate [44]. Hence, the genetic organization of *atf1* with the upstream and downstream genes was analyzed. Interestingly, *atf1* (*Mn_4963*) shows forms a putative gene operon with three downstream genes, including *Mn_4963*, *Mn_4964*, and *Mn_4965* (Additional file 1: Figure S2A). Further analyses showed that their transcriptional levels in strain WIII were significantly upregulated in the presence of sterols. At 72 h after 0.5 g/L phytosterols added, the transcription intensities of *atf1*, *Mn_4963*, *Mn_4964*, and *Mn_4965* were upregulated by 46-fold, 13.1-fold, 8.1-fold, 1.4-fold and 2.0-fold, respectively (Additional file 1: Table S1). The phenomenon suggested that all four genes are involved in the catabolism and may be related to the HBC sub-pathway. Therefore, the operon was named the HBC operon.

The putative HBC operon was located distal to the sterol degradation gene cluster in strain *Mn*. Gene annotation analyses indicated that *Mn_4963* encodes acyl dehydratase and *Mn_4964* encodes acyl-CoA dehydrogenase. According to the functional annotation and orthologs characterized in the catabolism of sterols, these genes were named *chsH4* and *chsE6*, respectively. Therefore, *chsH4*, *atf1* and *chsE6* were speculated to participate in a three-step sequential reaction from 3-oxo-chol-4,22-dien-24-oyl-CoA (3-OCDO-CoA) to 4-HBC (Additional file 1: Figure S2B). In addition, *Mn_4965* was annotated as a gene encoding a TetR/AcrR family transcriptional regulator. To date, two TetR/AcrR family transcriptional regulators, including KstR1 and KstR2, have been deciphered as key factors in regulating the uptake, side-chain degradation, and rings A/B and C/D degradation of sterols, respectively [45, 46]. This implied a unique regulatory role of *Mn_4965* in sterol catabolism, which was named *kstR3*. Since it is encompassed in the putative HBC operon, *kstR3* might be involved in the transcriptional regulation of the putative HBC pathway. Based on the above analyses and speculation, the functions of these genes in the putative HBC operon were elucidated.

### Functional identification of KstR3

**Fig. 4:**
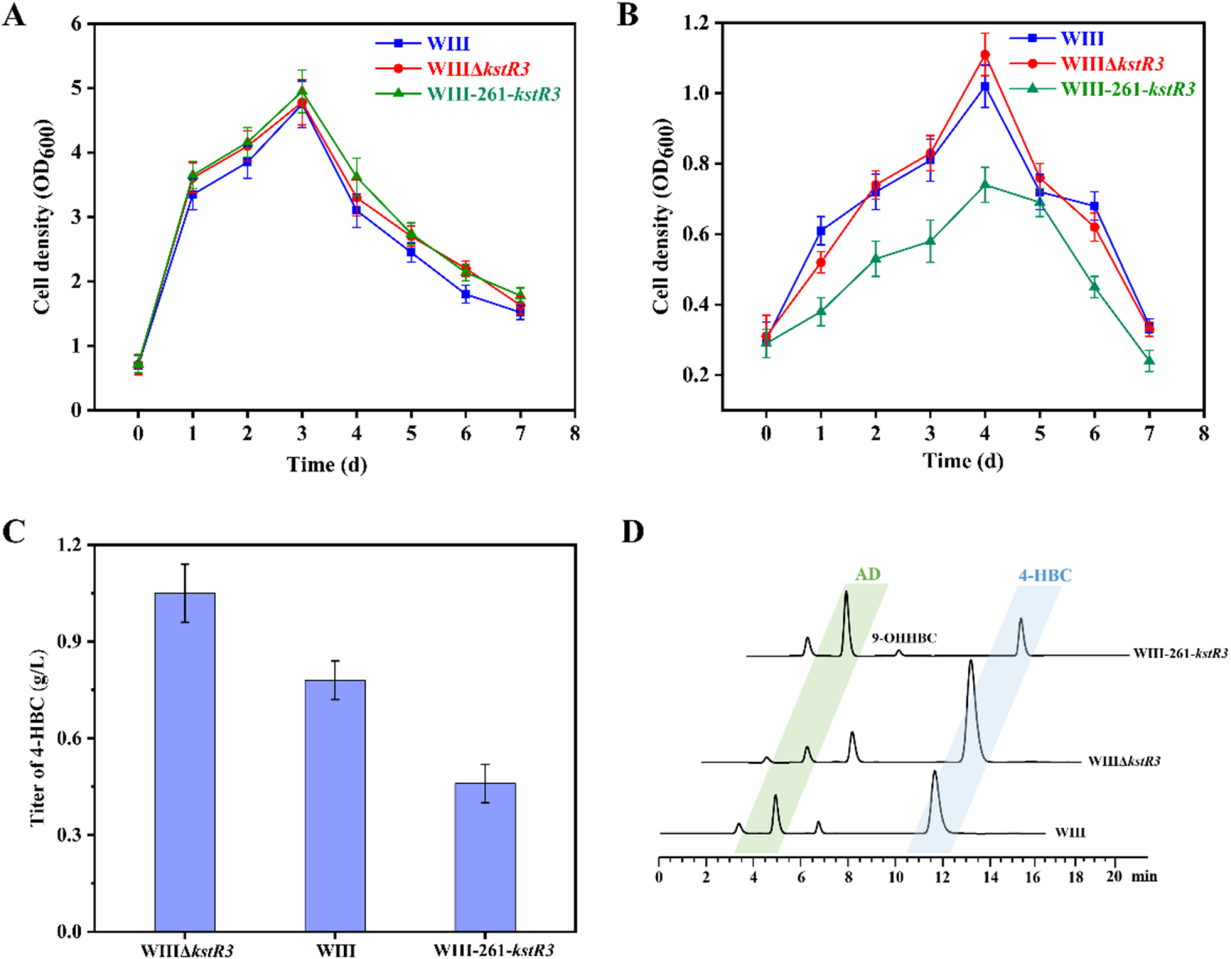
Effects of KstR3 on the conversion of phytosterols to 4-HBC in strain WIII. **A** The growth curves of strains WIII, WIIIΔ*kstR3* and WIII-261-*kstR3* in the MYC/01 medium containing glycerol. **B** The growth curves of strains WIII, WIIIΔ*kstR3* and WIII-261-*kstR3* in the MM medium with 1 g/L phytosterols as the sole carbon source. **C** 4-HBC production titers of strains WIII, WIIIΔ*kstR3*, and WIII-261-*kstR3*. **D** HPLC profiles of the phytosterols transformation by strains WIII, WIIIΔ*kstR3*, and WIII-261-*kstR3*. All assays were performed in triplicate with three independent measurements. Standard deviations of the biological replicates are represented by error bars

To determine its role in sterol catabolism, *kstR3* was knocked out and overexpressed in strain WIII to construct strains WIIIΔ*kstR3* and WIII-261-*kstR3*, respectively. First, the effect of *kstR3* was studied on the growth of strains. No significant growth differences were observed in strains WIII and WIIIΔ*kstR3* when they were cultured in either the MYC/01 medium containing glycerol or the minimum medium (MM) with phytosterols as the sole carbon source, while strain WIII-261-*kstR3* showed growth inhibition in the MM medium (Fig. 4A, B). This phenomenon could be explained by the fact that the HBC sub-pathway was blocked with the overexpression of *kstR3*, resulting in the restriction of the energy source. Subsequently, their performances on phytosterols transformation showed that compared to strain WIII, the 4-HBC production titer of strain WIIIΔ*kstR3* increased by 31.7%, while the 4-HBC titer of strain WIII/261-*kstR3* decreased by 73.6% (Fig. 4C). Moreover, AD production from the AD sub-pathway significantly decreased in strain WIIIΔ*kstR3* compared to WIII, whereas the titer of AD was correspondingly increased in strain WIII-261-*kstR3* (Fig. 4D). These results demonstrated a crucial regulatory role of *kstR3* in the competition between the AD and the HBC sub-pathway, i.e., KstR3 may exert a negative regulatory role on the HBC sub-pathway, which is similar to the regulatory mechanism of KstR1 and KstR2 on the main degradation gene cluster of sterols or it may exert a positive regulatory function on the AD sub-pathway. Therefore, the genes regulated by KstR3 were identified further.

Furthermore, the analysis of the transcriptomic data of strain WIIIΔ*kstR3* against strain WIII under phytosterols induction showed that seven genes were significantly altered at the transcriptional level (four upregulated and three downregulated) (Additional file 1: Table S2). Interestingly, the four upregulated genes were clustered in the genome from gene *Mn_4961* to *Mn_4965*, including the genes of the putative HBC operon, *atf1*, *chsH4* and *chsE6*, and the gene *Mn_4961* upstream of the putative HBC operon. Conversely, the three downregulated genes showed no clear correlation with sterol catabolism. Therefore, it could be deduced that KstR3 is a negative transcriptional regulator of the putative HBC operon. Strikingly, it is a negative regulatory factor of the HBC sub-pathway due to its negative regulatory effect on the transcription of *atf1*, the essential gene to initiate the HBC sub-pathway, and *chsE4* and *chsE6*, which might be the key genes involved in the HBC sub-pathway. This phenomenon was consistent with the above results that the production of 4-HBC was significantly enhanced in strain WIIIΔ*kstR3* against WIII because of the upregulated transcriptional levels of genes *atf1*, *chsH4* and *chsE6*.

### Role identification of ChsH4

**Fig. 5:**
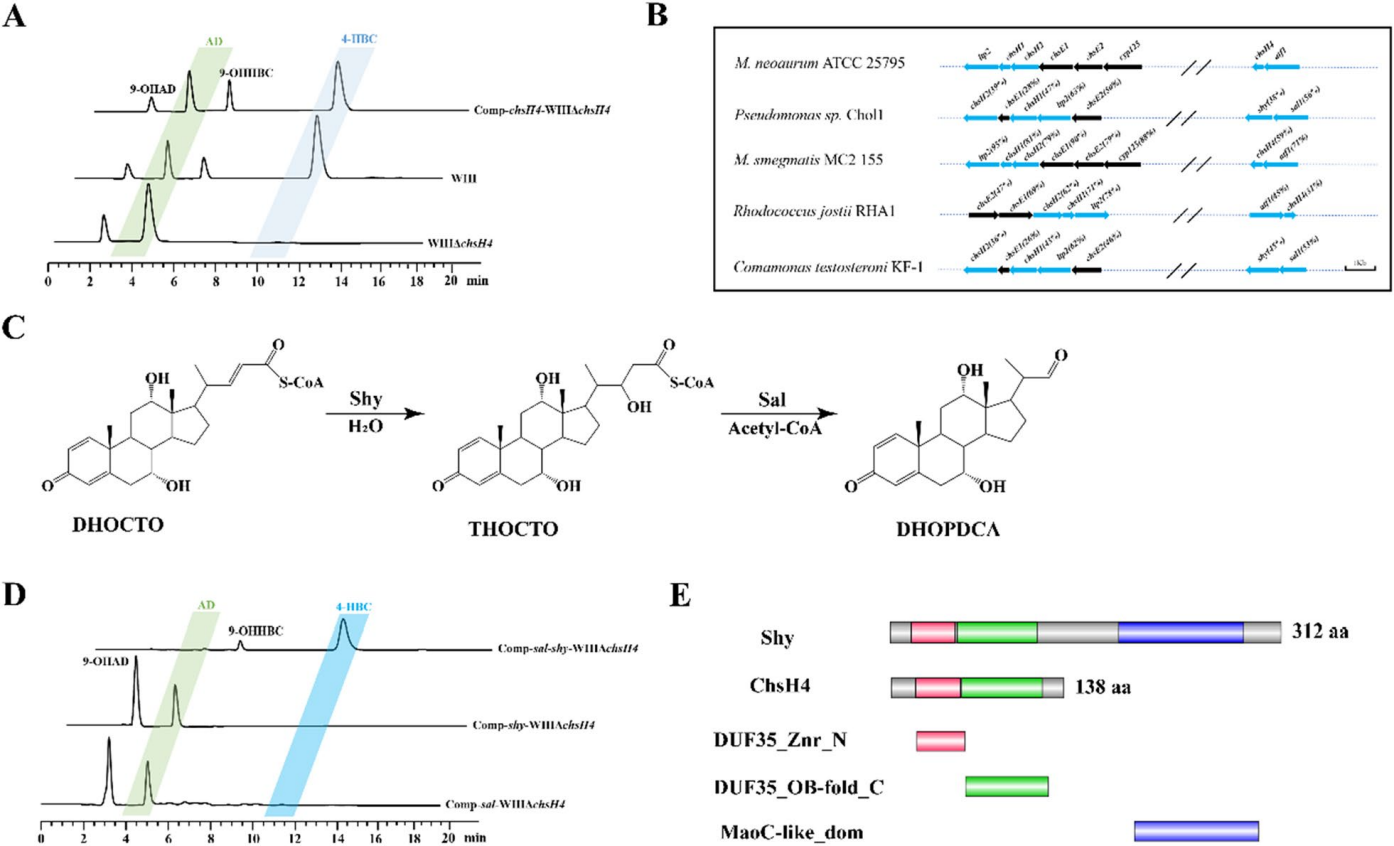
Functional identification of ChsH4. **A** HPLC profiles of strains WIII, WIIIΔ*chsH4*, and Comp-*chsH4*-WIIIΔ*chsH4* in the conversion of phytosterols. **B** Characteristic genomic distribution of thiolase/aldolase and hydrase genes in sterol-degrading microorganisms. Thiolase/aldolase and hydrase genes are marked in blue arrows, the other genes are marked in black arrows, the length and orientation of arrows represent the length and orientation of genes, the numbers in parentheses indicate the similarity in amino acid sequences. **C** Schematic of the catalytic mechanism of Sal1 and Shy. **D** HPLC profiles of strains WIIIΔ*chsH4*, Comp-*shy*-WIIIΔ*chsH4* and Comp-*sal*-*shy*-WIIIΔ*chsH4* in the transformation of phytosterols. **E** Analysis of the conserved domain between ChsH4 and Shy

To determine the role of *chsH4* in sterol catabolism, the deletion and complementation of *chsH4* were performed in strain WIII, generating strains WIIIΔ*chsH4* and Comp-*chsH4*-WIIIΔ*chsH4*, respectively. Compared to WIII, strain WIIIΔ*chsH4* showed significant growth inhibition in the MM medium with phytosterols as the sole carbon source (Additional file 1: Figure S3A), while it grew normally in the seed medium containing glycerol (Additional file 1: Figure S3B), suggesting that the utilization of phytosterols was inhibited in strain WIIIΔ*chsH4* due to the deletion of *chsH4*. Strikingly, the production of 4-HBC and its derivative 9-OHHBC was blocked, while the production of AD and 9-OHAD, the products of the AD sub-pathway, was not attenuated (Fig. 5A), indicating that *chsH4* is an essential gene in the putative HBC sub-pathway but has no direct correlation with the AD sub-pathway. The correlation between *chsH4* and the HBC sub-pathway was determined by the complementation of *chsH4* in strain Comp-*chsH4*-WIIIΔ*chsH4* (Fig. 5A).

*ChsH4* has been annotated as an acyl dehydratase-encoding gene and joined end-to-head to *atf1* on the genome. This genetic organization was common and conserved across the steroid-degrading strains (Fig. 5B), including the consecutive arrangement of *sa1l* and *shy* on the genome of *Pseudomonas* sp. Chol1. Considering that *sal1* is a counterpart of *atf1* and can replace *atf1* to exert a key role in the putative HBC sub-pathway, it was speculated that *chsH4* might also be a counterpart of *shy*. In addition, *shy* was identified to encode an enoyl-CoA hydratase (Shy) responsible for the hydration of 7α,12α-dihydroxy-3-oxochola-1,4,22-triene-24-oly-CoA (DHOCTO) to 7α,12α,22-trihydroxy-3-oxochola-1,4-diene-24-oate (THOCDO) (Fig. 5C). The previous reaction of the step was catalyzed by Sal1 in the side chain degradation pathway of cholate, i.e. (Fig. 3B) [44], similar to Shy, ChsH4 may act as an enoyl-CoA hydratase to catalyze the formation of 22-hydroxy-3-oxo-cholest-4-en-24-oyl-CoA (22-HOCO-CoA) from 3-OCDO-CoA (Additional file 1: Figure S2B). However, the expression of *shy* in strain Comp-*shy*-WIIIΔ*chsH4* could not restore the production phenotype of strain WIII (Fig. 5D), implicating that *shy* may not be the counterpart of *chsH4*.

In the catabolic process of aliphatic acids, the genes encoding hydratase and thiolase are often coupled to overcome the thermodynamic disadvantages of hydration for unhindered metabolism [47]. A similar phenomenon between *shy* and *sal1* had also been shown for the side chain degradation of cholate [44]. Thus, it could be inferred that *chsH4* and *atf1* are closely coupled in strain *Mn* for the side chain degradation of sterols. Thus, the finding that *chsH4* could not be functionally replaced by *shy* may be attributed to the fact that *shy* cannot be coupled in strain Comp-*shy*-WIIIΔ*chsH4*. Since *atf1* can be functionally replaced by *sal1* in strain Comp-*sal*-WIIIΔ*atf1* (Fig. 3A), *sal1* was further introduced into strain WIIIΔ*chsH4* with *shy*. Consequently, the generated strain Comp-*sal*-*shy*-WIIIΔ*chsH4* displayed the same metabolic phenotype as strain WIII (Fig. 5D), indicating that the deficiency of *chsH4* can be compensated in the function by the co-expression of *sal1* and *shy*. To exclude the possibility that recovery of metabolic phenotype was due to the expression of *sal1* alone, strain Comp-*sal*-WIIIΔ*chsH4* was constructed. No 4-HBC was detected in this strain during sterols transformation, which confirmed that the functional compensation of *chsH4* was attributed to the combined expression of *sal1* and *shy* (Fig. 5D). *Sal1* has been verified as a counterpart of *atf1*, indicating that the combination function of *atf1* and *chsH4* can be well replaced by *sal1* and *shy* couple, although *shy* is not the counterpart of *chsH4* in strain WIII.

To further reveal the differences in *chsH4* from *shy*, their coding sequences were analyzed. In terms of length, ChsH4 (138 amino acids) showed to be only 44% of Shy (312 amino acids), and the alignment analysis indicated that ChsH4 could only be matched to the N-terminal sequence of Shy (Additional file 1: Figure S4). Moreover, Shy consists of two conserved domains: DUF35 (domain of unknown function 35) at the N-terminus and MaoC-like domain at the C-terminus (Fig. 5E). Conversely, ChsH4 contains only the DUF35 domain. Since the catalytic activity center of Shy is in the MaoC-like domain, it can be affirmed that ChsH4 is not a functional hydratase and possesses only a partial function of Shy.

DUF35 is a common and conserved domain among some enzymes, such as thiolase, acyl-CoA dehydrogenases, short chain acyl-CoA dehydrogenases, and sterol transport proteins [48, 49]. However, its specific function is yet to be clarified. In a well-characterized pair of hydratase and thiolase involved in the side-chain degradation of sterols, Ltp2-ChsH1/H2, DUF35 domain is conserved at the C-terminal of ChsH2 [47, 50]. Additionally, DUF35 domain acts as a molecular staple mediating the formation of a bifunctional enzyme complex with the successive activity of hydratase and thiolase. Then, the DUF35 domain of ChsH2 forms a functional thiolase complex with Ltp2 [50]. As an independent DUF35 protein, ChsH4 may also act as a molecular staple to mediate the combination of a hydratase with Atf1 to catalyze a successive hydration and retro-aldol reaction from 3-OCDO-CoA to 3-OPA to overcome the thermodynamic disadvantages of the hydration of 3-OCDO-CoA to 22-OH-BNC-COA in the putative HBC sub-pathway. Nonetheless, the specific mechanism needs to be characterized further.

### Role analysis of ChsE6

**Fig. 6:**
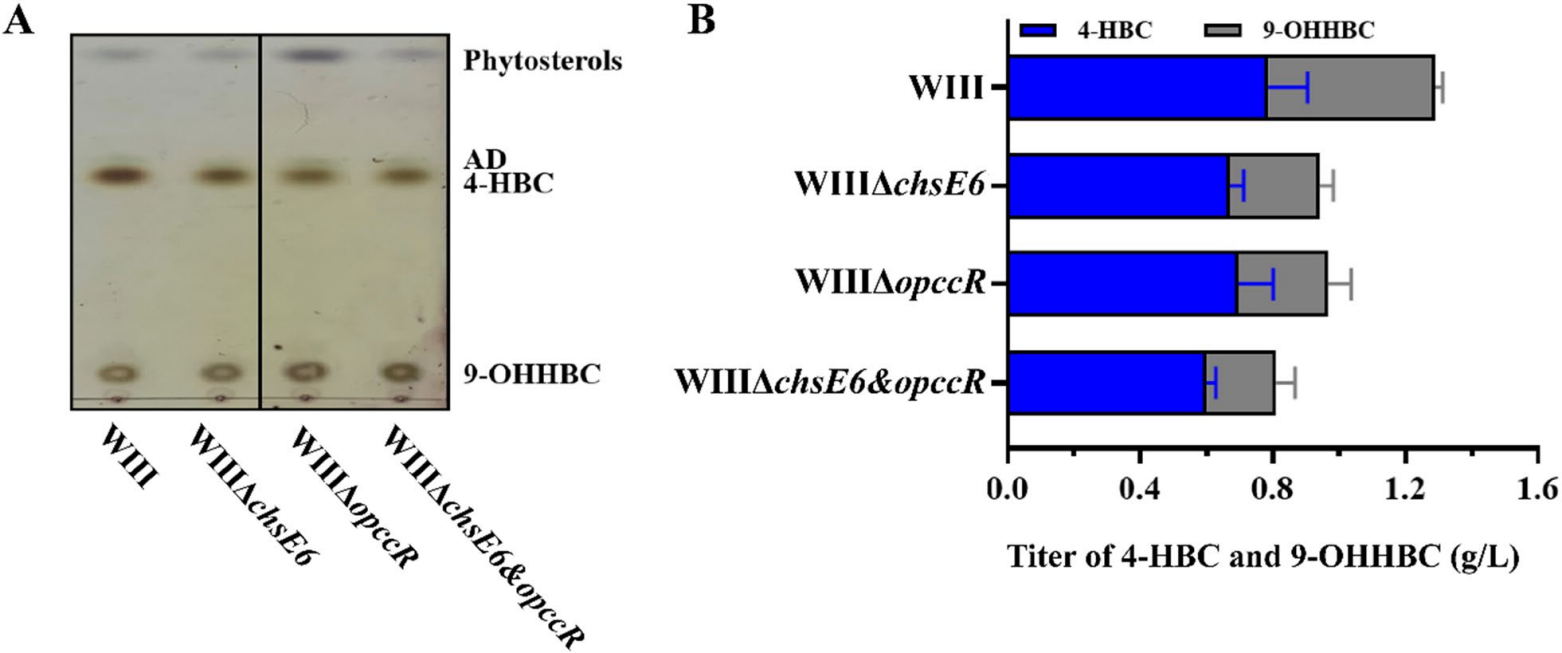
Functional analysis of ChsE6. **A** TLC chromatogram of the metabolite profiles of strains WIII, WIIIΔ*chsE6*, WIIIΔ*opccR*, and WIIIΔ*chsE6*&*opccR* in the conversion of phytosterols. **B** C-22 metabolite (4-HBC and 9-OHHBC) production titer of strains WIII, WIIIΔ*chsE6*, WIIIΔ*opccR*, and WIIIΔ*chsE6*&*opccR* in the transformation of phytosterols. All assays were performed in triplicate with three independent measurements. Standard deviations of the biological replicates are represented by error bars

In addition to the conversion of 22-OH-BNC-CoA to 3-OPA, there is also a conversion reaction from 3-OPA to 4-HBC in the proposed HBC sub-pathway. In the HBC operon, *chsE6* gene encodes the protein annotated as a dehydrogenase, and a significant positive correlation has been established between *chsE6* and 4-HBC production in transcriptomics and proteomics. Therefore, ChsE6 was presumed to be responsible for the reduction reaction of 3-OPA to 4-HBC. Then, strain WIIIΔ*chsE6* was constructed by knocking out *chsE6* in strain WIII. The results of phytosterols transformation showed that 4-HBC could be detected in strain WIIIΔ*chsE6* (Fig. 6A), although its combined production titer of 4-HBC and 9-OHHBC was reduced by 27% compared to strain WIII (Fig. 6B). This phenomenon confirmed that *chsE6* is closely related to the production of 4-HBC. However, the specific role of *chsE6* could not be identified. To directly determine the function of ChsE6, the conversion of 3-OPA to 4-HBC was effectuated by the heterogeneously expressed ChsE6. Nonetheless, no reaction was detected (data not shown), implying that *chsE6* may not be the key enzyme involved in the catalysis from 3-OPA to 4-HBC; this needs to be explored further.

Previously, *mnOpccR* from *M. neoaurum* CCTCC AB2019054 had been identified as a bifunctional reductase, which converted 3-OPC-CoA, the product of thiolase FadA5 in the AD sub-pathway to 4-HBC and reduced 3-OPA to 4-HBC via its N-terminal domain [40]. Hence, the ortholog of *mnOpccR*, *opccR* was knocked out in strain WIII. The generated strain WIIIΔ*opccR* showed a similar result to strain WIIIΔ*chsE6* in the conversion of phytosterols (Fig. 6A, B). Then, a double-knockout strain of *chsE6* and *opccR*, WIIIΔ*chsE6*Δ*opccR*, was constructed, which showed that the conversion capacity of phytosterols to 4-HBC was unblocked but attenuated (Fig. 6B). All these phenomena suggested that multiple enzymes may be involved in the reduction of 3-OPA to 4-HBC in the putative HBC sub-pathway.

### An updated strategy to develop improved strains for the conversion of phytosterols to steroidal synthons

The microbial catabolism of sterols is a sophisticated process, and some mechanisms remain obscure [14]. Although the main gene cluster for sterols degradation has been identified and considerable progress has been made in the use of metabolic engineering techniques to develop high-yielding strains for the conversion of phytosterols to valuable steroids [7, 21], the development of industrial strains faces some challenges, such as inevitable byproduct formation and low yields. For instance, 4-HBC is a common byproduct of the AD-producing strains, 9-OHHBC is a usual byproduct of the 9-OHAD-producing strains, and 9-OHAD is a familiar byproduct of AD-producing strains [51, 52]. Since the role of the HBC operon in the catabolic process of sterols has been revealed (Fig. 1), an updated measure can be designed to develop improved strains for the conversion of phytosterols to valuable products, such as 4-HBC, AD, and 9-OHAD, with lower byproducts and higher yields; for example, the development of an improved 4-HBC-producing strain as well as a 9-OHAD-producing strain using WIII and NwIB-I as starting strains, respectively.

Strain WIII is a 4-HBC-producing strain constructed previously by deleting some key genes involved in the degradation of steroid nucleus in *M. neoaurum* ATCC 25795, including *kshA1*, *hsd4A*, *kstD1*, *kstD2*, and *kstD3* (Table 1). The performance of strain WIII in sterols transformation indicated three main byproducts, AD, 9-OHAD, and 9-OHHBC, which severely reduces the yield of 4-HBC (Fig. 2A). Thus, to improve the performance of WIII, the generation of AD and 9-OHHBC should be blocked. According to the knowledge of the catabolic pathway of sterols (Fig. 1), an updated strategy was implemented to modify strain WIII.

**Fig. 7:**
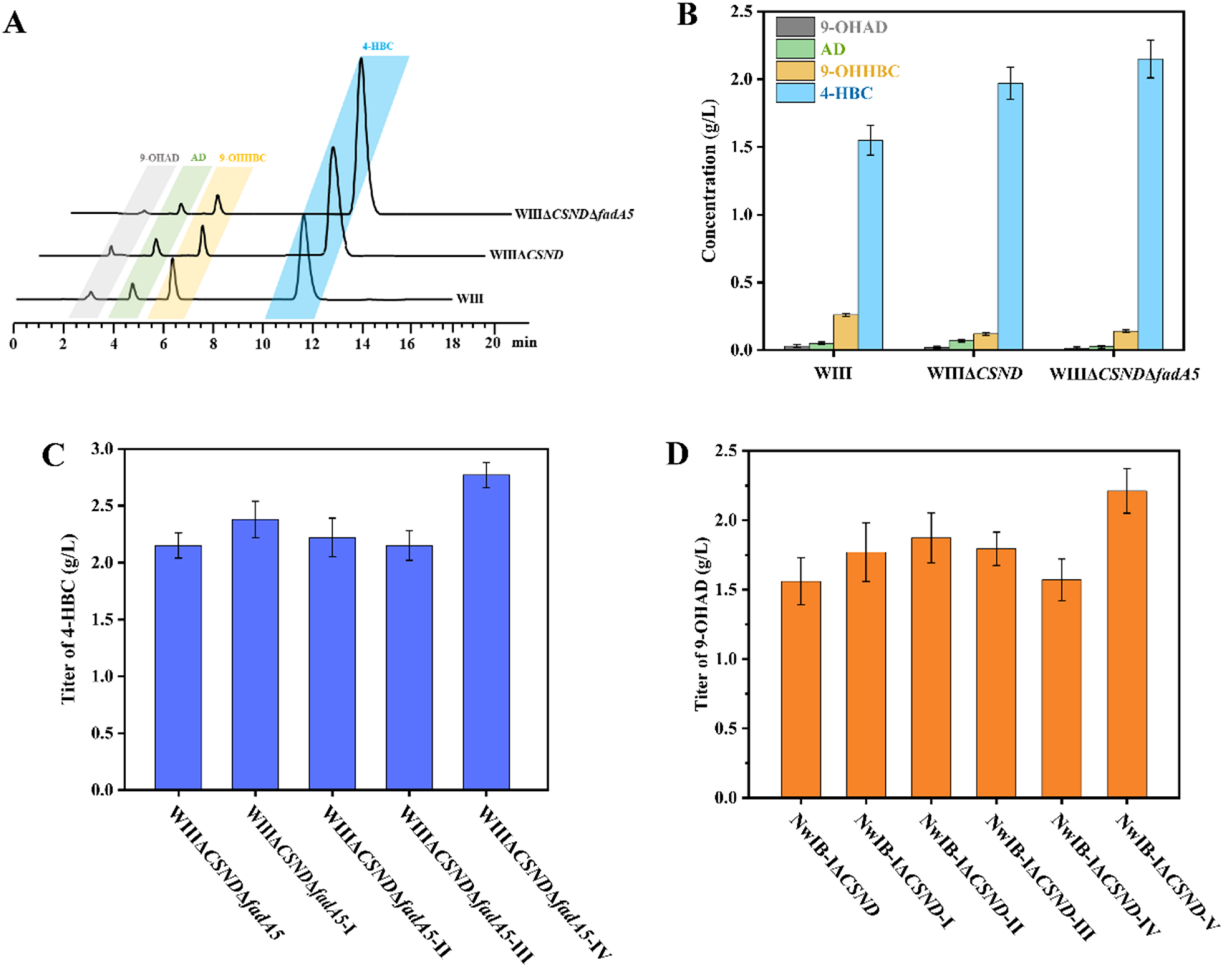
Construction of improved strains for the conversion of phytosterols into steroid synthons. **A** HPLC profiles of strains WIII, WIIIΔ*CSND*, and WIIIΔ*CSND*Δ*fadA5* in the transformation of phytosterols. **B** Production titers of strains WIII, WIIIΔ*CSND*, and WIIIΔ*CSND*Δ*fadA5* in the conversion of phytosterols. Effect of individual or combined over-expression of *atf1*, *chsH4*, and *chsE6* in strain WIIIΔ*CSND*Δ*fadA5* on the production of 4-HBC. **C** Effects of the individual or combined over-expression of *atf1*, *chsH4* and *chsE6* in strain WIIIΔ*CSND*Δ*fadA5* on the production of 4-HBC. **D** Effects of the individual or combined deletion of *atf1*, *chsH4*, *chsE6* or expression of *kstR3* in strain NwIB-IΔ*CSND* on the production of 9α-OHAD. All assays were performed in triplicate with three independent measurements. Standard deviations of the biological replicates are represented by error bars

The generation of AD, 9-OHAD, and 9-OHHBC indicated some activities of 3-ketosteroid-9α-hydroxylase (Ksh) and the AD sub-pathway in strain WIII, although the main Ksh encoding gene *kshA1* and the key gene *hsd4A* involved in the AD sub-pathway have been inactivated in strain WIII [18]. Therefore, the remaining activities of Ksh and the AD sub-pathway were attenuated by deleting the homologs of *kshA1* and the key gene *fadA5* involved in the AD sub-pathway. Firstly, genome analysis showed that besides *kshA1* there is still a gene encoding a putative homolog of *kshA* (designated as *kshA2*). Intriguingly, *kshA2* is close to *kstD2* and *kstD3* on the genome, which have been deleted in strain WIII due to their critical roles in steroid nucleus degradation. Their genetic organization is highly conserved across *Mycolicibacteria* [7, 53], which has been defined as a cryptic cluster of steroid nucleus degradation (CSND) in *M. smegmatis* (Additional file 1: Figure S5A, B). Therefore, to block the activities of Ksh and other enzymes involved in the degradation of steroid nucleus, strain WIIIΔ*CSND* was constructed by deleting CSND in strain WIII. The performance of strain WIIIΔ*CSND* in the conversion of phytosterols displayed that the total amount of 9-OHAD and 9-OHHBC decreased by 51.7%, while the production of 4-HBC increased by 27.1% to 1.97 g/L (Fig. 7A, B, Additional file 1: Figure S5C). A similar phenomenon was observed in strain NwIB-IΔ*CSND*, which showed a 26.7% increase of 9-OHAD in contrast to strain NwIB-I developed previously by deleting *kstD1*, *kstD2* and *kstD3* genes (Additional file 1: Figure S5D) [52]. Secondly, gene *fadA5* was deleted in strain WIIIΔ*CSND* to further reduce the residual activity of the AD sub-pathway, generating strain WIIIΔ*CSND*Δ*fadA5*. Compared to that in strain WIIIΔ*CSND*, the total content of byproducts AD and 9-OHAD decreased by 56.7%, and the yield of 4-HBC was increased by 9.14% to 2.15 g/L in strain WIIIΔ*CSND*Δ*fadA5* (Fig. 7A, B). These results confirmed that the performance of the engineered strains for the conversion of phytosterols to steroid synthons can be significantly improved by modifying the well-characterized pathways of steroid nucleus degradation and the AD sub-pathway. Nevertheless, AD cannot be completely removed from the 4-HBC-producing strain, and 9-OHHBC is not capable of removal from the 9-OHAD-producing strain, which could be attributed to the correlation between the AD and the HBC sub-pathways.

**Fig. 8:**
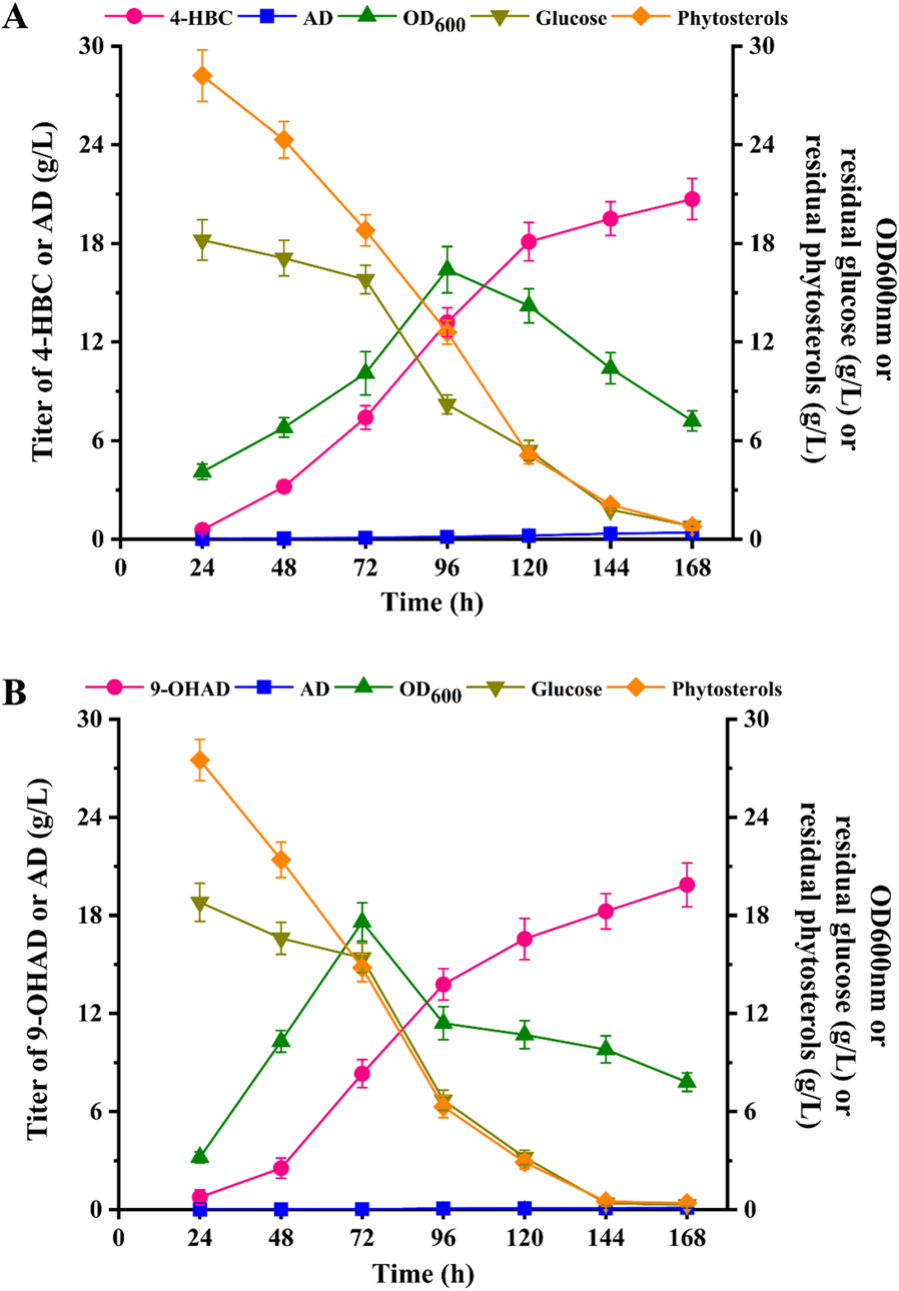
Time course of the conversion of 30 g/L phytosterols by strains WIIIΔ*fadA5*Δ*CSND*-IV (**A**) and NwIB-IΔ*CSND*-V (**B**) in a 5-L bioreactor. All assays were performed in triplicate with three independent measurements. Standard deviations of the biological replicates are represented by error bars

Owing to the coexistence of AD and HBC sub-pathways, reducing the competition of the production of AD-type byproducts on the production of HBC-type products in the engineered *Mycolicibacteria* is challenging when some key mechanisms involved in the HBC sub-pathway are unclear, and vice versa. In the present study, the key operon involved in the HBC sub-pathway offers a new solution for these difficulties. Based on the above engineered strain WIIIΔ*CSND*Δ*fadA5*, the individual and combined overexpression of *atf1*, *chsH4* and *chsE6* from the HBC operon was utilized to determine the effects of the HBC sub-pathway on the production of 4-HBC in the engineered strains. Compared to strain WIIIΔ*CSND*Δ*fadA5*, the 4-HBC titer of strains WIIIΔ*CSND*Δ*fadA5*-I and WIIIΔ*CSND*Δ*fadA5*-II was increased by 10.9% and 3.3% (Fig. 7C), and the amounts of AD decreased by 40.7% and 16.4%, respectively (Additional file 1: Figure S6A), whereas no obvious change was observed in the metabolic phenotype of strain WIIIΔ*CSND*Δ*fadA5*-III (Fig. 8C). These results confirmed the key roles of *atf1* and *chsH4* in the HBC sub-pathway, and determined *atf1* as the main rate-limiting gene for the production of HBC-type products. Therefore, *atf1* and *chsH4* was overexpressed together in strain WIIIΔ*CSND*Δ*fadA5*. Subsequently, the generated strain WIIIΔ*CSND*Δ*fadA5*-IV showed a 28.9% increase in 4-HBC titer and a 56.4% decrease in AD titer (Additional file 1: Figure S6A). These data suggested that the metabolic flow of the HBC sub-pathway can be efficiently augmented by over-expressing *atf1* and *chsH4*. Therefore, the strategy to modify the conversion pathway from sterols to a specific valuable product can be updated by adding the modifications on the HBC sub-pathway, including augmenting its activity for the production of HBC-type products and blocking its activity for the production of AD-type products.

Next, the applicability of strain WIIIΔ*CSND*Δ*fadA5*-IV in the production of 4-HBC was evaluated by a scale-up test in a 5-L fermenter with an increasing feed concentration of phytosterols up to 30 g/L. Due to the inhibitory effect of high concentration on strain growth, phytosterols were added into the fermentation media after 10 h cultivation of strain WIIIΔ*CSND*Δ*fadA5*-IV. The process of fermentation was terminated at 168 h when the substrate concentration could not be altered at 0.78 g/L. Finally, the titer of 4-HBC reached 20.7 g/L with a conversion of 92.5%, and the titer of byproduct AD was 0.41 g/L (Fig. 8A, Additional file 1: Figure S6B). Compared to the documented 4-HBC-producing strains (Table 2), strain WIIIΔ*CSND*Δ*fadA5*-IV displayed an optimal production titer and byproduct content, indicating a better application prospect in the industry.

**Table 2:** Performances of different documented strains for 4-HBC production

Strains	Substrates (g/L)	Titer (g/L)	Refs.
*Mycobacterium* sp. NRRLB-11389	Cholesterol (10)	0.44	[54]
*M. neoaurum* DSM 1381Δ*kstD1*	Phytosterols (20)	14.18	[55]
*M. neoaurum* XIIΔ*hsd4A*Δ*kstD123*	Phytosterols (40)	5.24–5.75	[18]
*M. neoaurum* WIIIΔ*mmpL3*-*cyp125*-*choM1*-*fadA5*	Phytosterols (20)	7.59	[56]
*M. neoaurum* WIIIΔ*mmpL3*-*pbpB*	Phytosterols (20)	6.66	[57]
*M. neoaurum* WIII-*egt*&*msh*&*cat*	Phytosterols (20)	12.5	[9]
*M. neoaurum* WIIIΔ*kasB*	Phytosterols (20)	7.8	[10]
*M. neoaurum* mJTU8	Phytosterols (20)	7.41	[40]
*M. neoaurum* WIIIΔ*CSND*Δ*fadA5*-IV	Phytosterols (30)	20.7	This work

In addition, an updated strategy was tested to develop an improved 9-OHAD- producing strain based on strain NwIB-IΔ*CSND* by modifying the genes of the HBC operon to transfer the metabolic flow from the HBC sub-pathway to the AD sub-pathway. Specifically, *kstR3* was overexpressed and *atf1*, *chsH4*, and *chsE6* were deleted in strain NwIB-IΔ*CSND*, generating strains NwIB-IΔ*CSND*-I, NwIB-IΔ*CSND*-II, NwIB-IΔ*CSND*-III and NwIB-IΔ*CSND*-IV, respectively. Compared to NwIB-IΔ*CSND*, the production titer of 9-OHAD in strains NwIB-IΔ*CSND*-I, NwIB-IΔ*CSND*-II, and NwIB-IΔ*CSND*-III was increased by 13.2%, 20% and 15%, respectively, while no obvious change was detected in strain NwIB-IΔ*CSND*-IV (Fig. 7D). This confirmed the negative regulatory role of *kstR3* and the key functional roles of *atf1* and *chsH4* in the HBC sub-pathway. Additionally, disrupting the function of the HBC operon is conducive to transferring the metabolic flow from the HBC to the AD pathway and enhancing the production of C-19 type steroids, such as 9-OHAD. Therefore, the strain NwIB-IΔ*CSND*-V was further constructed by deleting the HBC operon to block the HBC sub-pathway. Intriguingly, 2.21 g/L of 9-OHAD was achieved by strain NwIB-IΔ*CSND*-V, and its production titer was increased by 41.7% compared to that of strain NwIB-IΔ*CSND* (Fig. 7D). The performance of strain NwIB-IΔ*CSND*-V was further evaluated in a 5-L fermenter. Finally, the production titer of 9-OHAD reached up to 19.87 g/L with a 94.6% molar yield, and almost no by-products could be detected, indicating a good application prospect in the commercial production of 9-OHAD (Fig. 8B. Additional file 1: Figure S6C).

In summary, the above results confirmed the effectiveness of the updated strategy to develop improved strains producing C-19 and C-22 steroids with enhanced titer and yield and decreased by-product content by modifying the HBC operon involved in the HBC sub-pathway.

## Conclusions

In this study, we have discovered and identified a key operon involved in the HBC sub-pathway, which contains four key genes responsible for the metabolic transformation and regulation of the HBC sub-pathway, including an aldolase gene *atf1*, a DUF35-type gene *chsH4*, a reductase gene *chsE6*, and a negative transcriptional regulation gene *kstR3*. This finding not only confirms the putative HBC sub-pathway proposed in our previous study, but also opens up the possibility of further modification to improve the performances of the C-19 and C-22 steroid-producing strains. Therefore, an updated strategy was proposed to develop improved C-19 or C-22 steroid-producing stains by modifying the AD and HBC sub-pathways simultaneously. Taken together, this study not only revealed a key metabolic mechanism missing from the transformation of sterols in *Mycolicibacteria*, but also provided an updated way to develop improved *Mycolicibacteria* for the conversion of cheap phytosterols to valuable steroid synthons by metabolic engineering.

## Materials and methods

### Strains, plasmids, chemicals, and culture conditions

All strains used in this study are listed in Table 1. *Escherichia coli* DH5α (TIANGEN Biotech. Co., Ltd. Shanghai, China) was used for plasmid amplification. The wild type *Mycolicibacterium neoaurum* ATCC 25795 (*Mn*) as the source of identified genes was purchased from American Type Culture Collection (ATCC). A typical 4-HBC producing strain, named strain WIII, was constructed by deleting *kshA*, *hsd4A*, *kstD1*, *kstD2* and *kstD3* in strain *Mn*.^18^ A typical 9-OHAD producing strain, termed strain NwIB-I, was constructed by *kstD1*, *kstD2*, *kstD3* in strain *Mn*.^52^ The plasmids and primers used for the construction of engineering strains are described in Additional file 1: Table S3.

As a host for the molecular cloning of candidate genes, *E. coli* DH 5α was cultured at 37 °C and 220 rpm in liquid Luria–Bertani (LB) broth medium containing 50 mg/L kanamycin for plasmid selection. The culture condition of *Mycobacterium* was described according to previous studies.^54^ Briefly, *Mycobacterium* was firstly cultivated in 5 mL of LB until OD_600_ was 1.5. The strains were then transferred into the MYC/01 medium (glycerol 20 g/L, ammonium iron citrate 0.05 g/L, citric acid 2 g/L, MgSO_4_·7H_2_O 0.5 g/L, KNO_3_ 2.52 g/L, and (NH_4_)_2_HPO_4_ 1.65 g/L, pH 7.5). Then, fresh seeds were transferred into the MYC/02 medium (glucose 10 g/L, ammonium iron citrate 0.05 g/L, citric acid 2 g/L, MgSO_4_·7H_2_O 0.5 g/L, (NH_4_)_2_HPO_4_ 3.5 g/L, and K_2_HPO_4_ 0.5 g/L) with the addition of phytosterols. Before sterol conversion, phytosterol (100 g/L) was emulsified in Tween 80 (2.5%, w/v) aqueous solution at 121 °C for 1 h. The strain *Mycolicibacterium* was cultivated under the conditions of 30 °C and 200 rpm. 100 mg/L hygromycin or 50 mg/L kanamycin was used as the selection of *Mycolicibacterium* transformants.

The mixture of phytosterols was purchased from Davi Biochemistry (Zhejiang, China). Hydroxypropyl-β-cyclodextrin (HP-β-CD) was obtained from Shanghai Yuanye Biotechnology Co. Ltd. All other relevant reagents and substrates were prepared as previously reported [54].

### Construction of recombinant strains

The knockout strains were constructed through allelic homologous recombination in mycobacteria as previously described [14] p2NIL and pGOAL19 were used for the construction of the homologous recombination plasmids. To delete gene *atf1*, two fragments on both sides of the open-reading frame of *atf1* were amplified. Then, they were severally digested with restriction endonucleases (*Bam*H I/*Hin*d III), and ligated with the linearized p2NIL to acquire p2N-*atf1*. Finally, the selection marker cassette from pGOAL19 was inserted into the *Pa*c I site of p2N-*atf1* to generate the suicide plasmid pKO-*atf1*. Other plasmids, such as pKO-*fadA5*, pKO-*ltp2* and so on, were constructed in the same way. The plasmids were then electroporated into *Mycolicibacterium*, and the target gene deficient strains could be obtained following the two-step screening process.

To complement the deficient-gene function, the recombinant plasmid p261-*atf1* was firstly constructed, which could be used to overexpress the carried *atf1* in multiple copies. After the recombinant plasmids were verified by PCR, the correct plasmids were electro-transformed into *Mycolicibacterium* to generate the corresponding gene augmentation strains. Meanwhile, a complemental plasmid p306-*atf1* was obtained by integrating the expression cassette of the target *atf1* containing a heat shock promoter *hsp60* into the plasmid pMV306. The plasmid p306-*atf1* was then electro-transferred into *Mycolicibacterium* to obtain complemental strains, which was used to confirm the biofunction of *atf1* in vivo. The construction of other complemental strains underwent the same process [13].

### Sterol transformation analysis

The analysis of metabolites generated in the transformation of phytosterols was carried out according to our previous work.^9^ The samples were taken every 24 h during the process of phytosterol transformation and extracted with twice the volume of ethyl acetate. The supernatant was obtained and then dried under vacuum. Subsequently, the sample was re-dissolved in methanol for quantitative analysis, which was carried out by HPLC (Agilent Technologies, Inc., Santa Clara, CA, USA) on a SDB C18 column (5 μm, 4.6 mm × 250 mm, Elite, China) at 30 °C with methanol/water (80:20, v/v) as the mobile phase with a flow rate of 1 mL/min. All metabolites were detected by an ultraviolet spectrophotometry at 254 nm. The remaining content of phytosterols was detected by gas chromatograph (GC) system (Agilent 7820 A, CA, USA) equipped with an HP-5 column (30 m × 0.25 mm, 0.25 μm film thickness). The oven temperature was programmed as follows: 200 °C for 2 min, 200 °C to 280 °C within 6 min, 280 °C for 3 min, 280 °C to 305 °C within 3 min, and 305 °C for 10 min. Inlet temperature and flame-ionization detector temperature were maintained at 320 °C. In addition, the formation of products was monitored by thin-plate chromatography (TLC) with HSGF254 plates (20 × 20 cm, Qingdao Marine Chemical Factory, China), and the petroleum ether/ethyl acetate (8: 2; v/v) was used as the developing solvent.

### Up-tank scale-up experiment of phytosterols conversion by engineering strains

The engineered strain was inoculated directly into MYC/01 seed medium and incubated at 30 °C and 220 rpm in a shaker until OD_600_ was approximately to 4, which was then transferred to MYC/01 seed medium under the same culture conditions. Notably, the OD_600_ could be measured only when the broth was extracted by ethyl acetate due to the hydrophobicity of the substrate phytosterols. When OD_600_ reached 4, the seed solution was inoculated into 5-L fermenter (10%, v/v) containing MYC/02 medium. After 8 h, the phytosterols were emulsified by cyclodextrin and added to the fermenter to a final concentration of 15 g/L, and the total volume of fermentation broth was 3.5 L. The initial speed of fermenter was 250 rpm and the air flow rate was set at 2 L/min, which were adjusted appropriately according to later fermentation. Samples were taken at 24 h intervals during the phytosterols transformation and the fermentation was monitored by TLC. The fermentation was terminated until the substrate was exhausted.

### Bioinformatics analysis

The genome information of *M. neoaurum* ATCC 25795 was sequenced and uploaded to NCBI in our previous work [52]. The transcriptomics and proteomics of mutant strains were determined and relevant analyses were completed. It was considered to be significant when the difference multiple exceeded 1.2 at protein abundance level and the value of *P* was lower than 0.05. The transcriptomics of strains WIIIΔ*kstR3* and WIII were completed by Shanghai Meguiar's Biomedical Technology Co. The analysis of transcriptomics and operon prediction were carried out by the online toolkit from Meguiar's Scientific Services (https://cloud.majorbio.com) and the software Rockhopper. Sequence alignment and phylogenetic analysis were performed by DNAMAN7 and MEGA5.1. The conserved domains of protein or coding nucleotide sequence were conducted at the NCBI website (https://www.ncbi.nlm.nih.gov/Structure/cdd/wrpsb.cgi). All data analyses were repeated at least 3 times. The data analysis of means ± standard deviation (SD) was completed with Origin 8 in this study.

## Supplementary Information

**Additional file 1: Figure S1.** Bioinformatics analysis of *Mn*_Atfs and their orthologous from typical sterol-consuming strains. **Figure S2.** Schematic of the genomic organization and the proposed catalytic mechanism of the putative HBC operon in *M. neoaurum* ATCC 25795. **Figure S3.** Effects of ChsH4 on the utilization of phytosterols in strain WIII. **Figure S4.** Comparison of secondary structures between ChsH4 and Shy. **Figure S5.** Genomic distribution and effect of CSND gene cluster. **Figure S6.** Analysis of metabolite profiles derived from sterols conversion by 4-HBC and 9-OHAD-producing strains. **Table S1.** Transcriptional changes of the genes flanking *atf1*. **Table S2.** Differential expression of genes in strains WIII and WIIIΔ*kstR3*. **Table S3.** Plasmids and primers used in this study.

## Data Availability

All data generated or analyzed during this study are included in this published article and its additional information files.

## Abbreviations

AD: Androstane-4-ene-3,17-dione
9-OH-AD: 9-Hydroxyandrost-4-ene-3,17-dione
4-HBC: 22-Hydroxy-23,24-bisnorchol-4-ene-3-one
22-OH-BNC-CoA: 22-Hydroxy-3-oxo-cholest-4-ene-24-carboxyl-CoA
3-OPA: 3-Oxo-4-pregnene-20-carboxyl aldehyde
Atf: Acetyl-CoA acetyltransferases
3-OCDO-CoA: 3-Oxo-chol-4,22-dien-24-oyl-CoA
DUF35: Domain of unknow function 35
CSND: Cluster of steroid nucleus degradation
