# Supplementary material for: Unravelling and engineering an operon involved in the side-chain degradation of sterols in Mycolicibacterium neoaurum for the production of steroid synthons
Source: Biotechnol Biofuels Bioprod. 2023 Aug 2;16:121. doi: 10.1186/s13068-023-02376-2 (PMC10398937; doi:10.1186/s13068-023-02376-2)
Supplement: Supplementary file 1 — Additional file 1: Figure S1. Bioinformatics analysis of Mn_Atfs and their orthologous from typical sterol-consuming strains. Figure S2. Schematic of the genomic organization and the proposed catalytic mechanism of the putative HBC operon in M. neoaurum ATCC 25795. Figure S3. Effects of ChsH4 on the utilization of phytosterols in strain WIII. Figure S4. Comparison of secondary structures between ChsH4 and Shy. Figure S5. Genomic distribution and effect of CSND gene cluster. Figure S6. Analysis of metabolite profiles derived from sterols conversion by 4-HBC and 9-OHAD-producing strains. Table S1. Transcriptional changes of the genes flanking atf1. Table S2. Differential expression of genes in strains WIII and WIIIΔkstR3. Table S3. Plasmids and primers used in this study. [file 13068_2023_2376_MOESM1_ESM.docx]

**Supplementary Information**

**Unravelling and engineering an operon involved in the side-chain degradation of sterols in *Mycolicibacterium neoaurum* for the production of steroid synthons**

Yun-Qiu Zhao^1^, Yong-Jun Liu^1^, Lu Song^1^, Dingyan Yu^1^, Kun Liu^1^, Ke Liu^1^, Bei Gao^1^, Xin-Yi Tao^1^, Liang-Bin Xiong^1, 2^, Feng-Qing Wang^1,^ * and Dong-Zhi Wei^1^

^1^State Key Laboratory of Bioreactor Engineering, Newworld Institute of Biotechnology, East China University of Science and Technology, 130 Meilong Road, Shanghai, 200237, China

^2^ Shanghai Key Laboratory of Molecular Imaging, Shanghai University of Medicine and Health Sciences, Shanghai 201318, China.

* Corresponding author:

* E-mail: fqwang@ecust.edu.cn, Tel: +86-21-6425-3287. Fax: +86-21-6425-0068. ORCID: 0000-0002-3473-5991.

**Contents of supplementary material:**

**Figure S1.** Bioinformatics analysis of *Mn*_Atfs and their orthologous from typical sterol-consuming strains.

**Figure S2.** Schematic of the genomic organization and the proposed catalytic mechanism of the putative HBC operon in *M. neoaurum* ATCC 25795.

**Figure S3.** Effects of ChsH4 on the utilization of phytosterols in strain WIII.

**Figure S4.** Comparison of secondary structures between ChsH4 and Shy.

**Figure S5.** Genomic distribution and effect of CSND gene cluster.

**Figure S6.** Analysis of metabolite profiles derived from sterols conversion by 4-HBC and 9-OHAD-producing strains.

**Table S1.** Transcriptional changes of the genes flanking *atf1*.

**Table S2.** Differential expression of genes in strains WIII and WIIIΔ*kstR3*.

**Table S3.** Plasmids and primers used in this study.


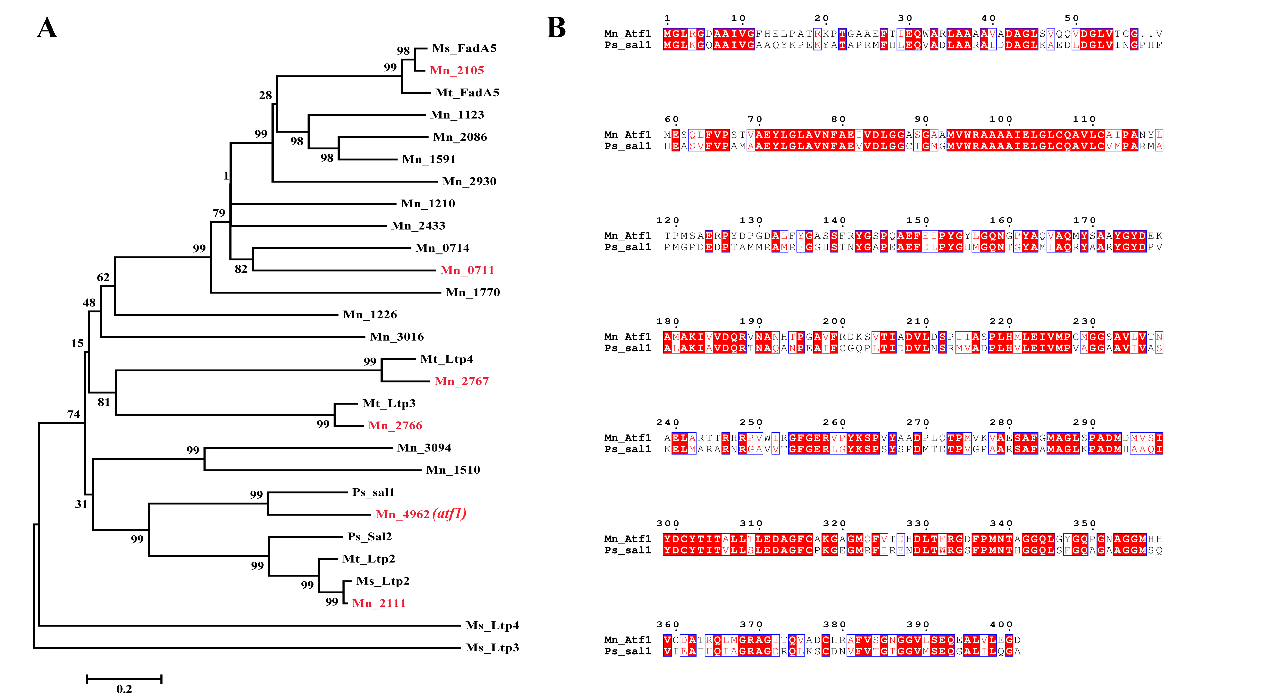


**Figure S1.** Bioinformatics analysis of *Mn*_Atfs and their orthologous from typical sterol-consuming strains. (A) Phylogenetic analysis of Atf1 and their orthologs. (B) Amino acid sequence alignment between Atf1 and Sal1, Query1 represents Sal1, Sbjct1 represents Atf1.


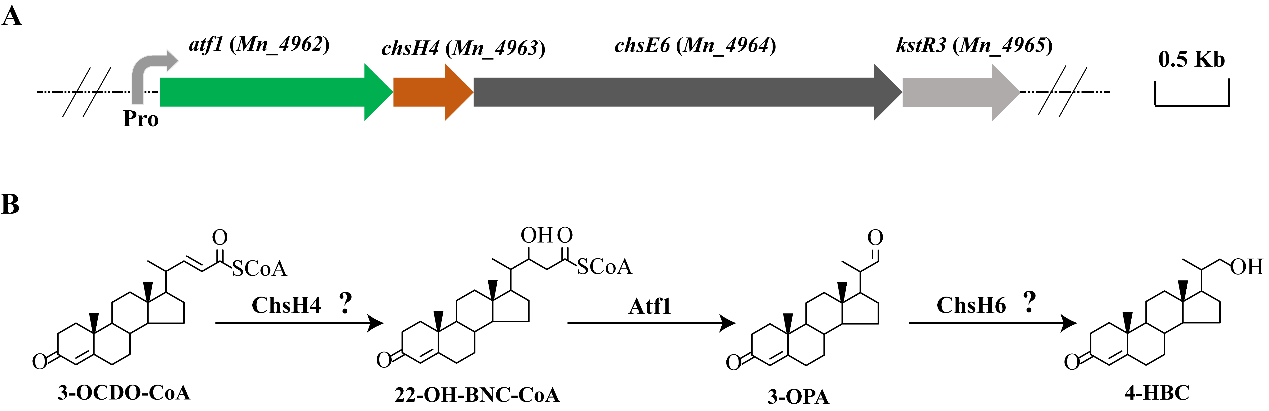


**Figure S2.** Schematic of the genomic organization (A) and the proposed catalytic mechanism (B) of the putative HBC operon in *M. neoaurum* ATCC 25795. The direction and sizes of the genes are indicated by arrows; and the spaces between the arrows indicate the distances between genes.


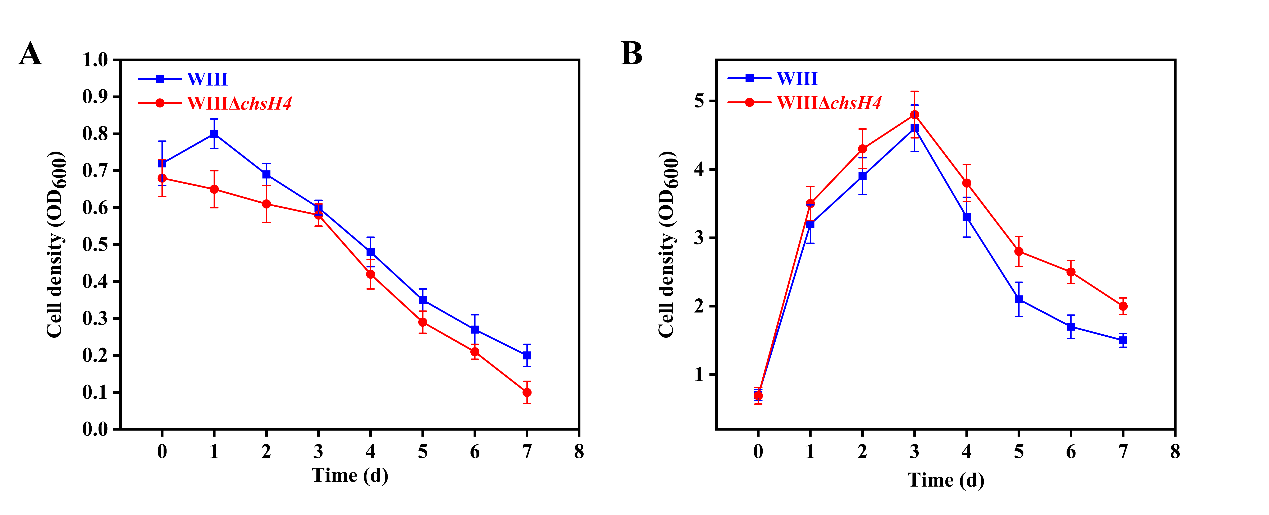


**Figure S3.** Effects of ChsH4 on the utilization of phytosterols in strain WIII. (A) The growth curves of strains WIII and WIIIΔ*chsH4* in the MM medium with 1 g/L phytosterols as the sole carbon source. (B) The growth curves of strains WIII and WIIIΔ*chsH4* in the MYC/01 medium containing glycerol. All assays were performed in triplicate with three independent measurements. Standard deviations of the biological replicates are represented by error bars.


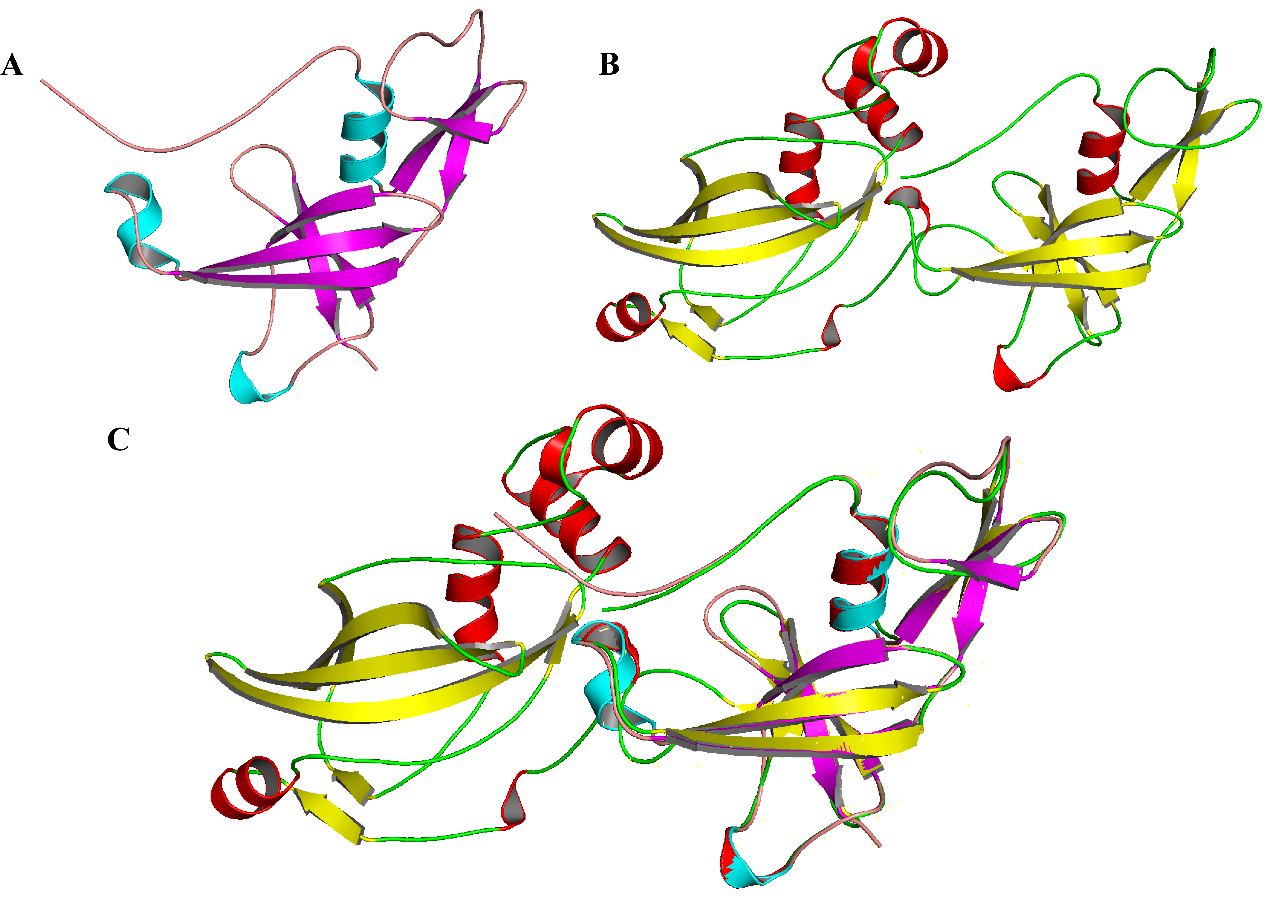


**Figure S4.** Comparison of secondary structures between ChsH4 and Shy. (A) Secondary structure of protein ChsH4. (B) Secondary structure of protein Shy. (C) Alignment of secondary structure of protein ChsH4 and Shy.


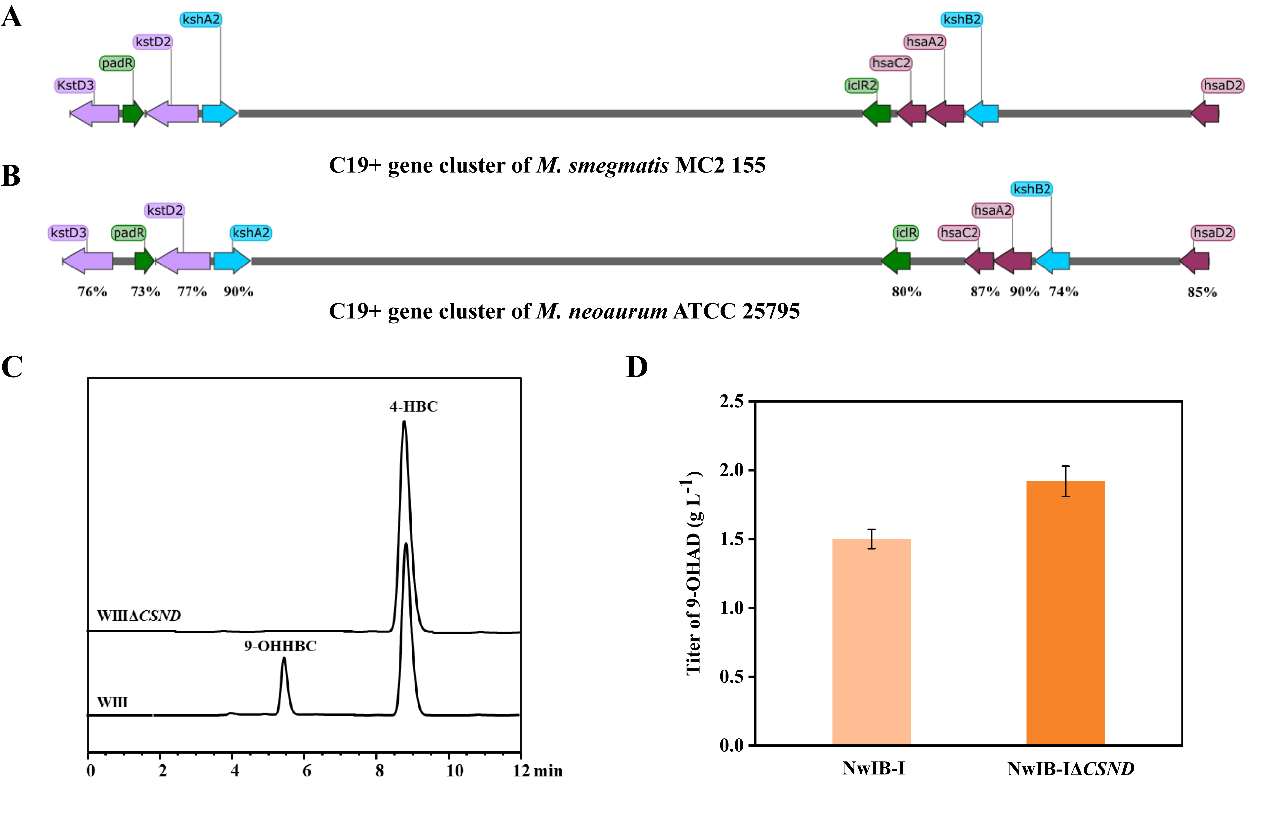


**Figure S5.** Genomic distribution and effect of CSND gene cluster. (A) Genomic distribution of the CSND gene cluster in *M. neoaurum* ATCC 25795. (B) Genomic distribution of the CSND gene cluster in *M. smegmatis* MC2 155. (C) Effects of CSND gene cluster on 4-HBC production. (D) Effects of CSND gene cluster on 9-OHAD production. The direction of the arrows indicated the orientation of genes, and the number blow the arrow represented the amino acid similarity of the two homologues. All assays were performed in triplicate with three independent measurements. Standard deviations of the biological replicates are represented by error bars.


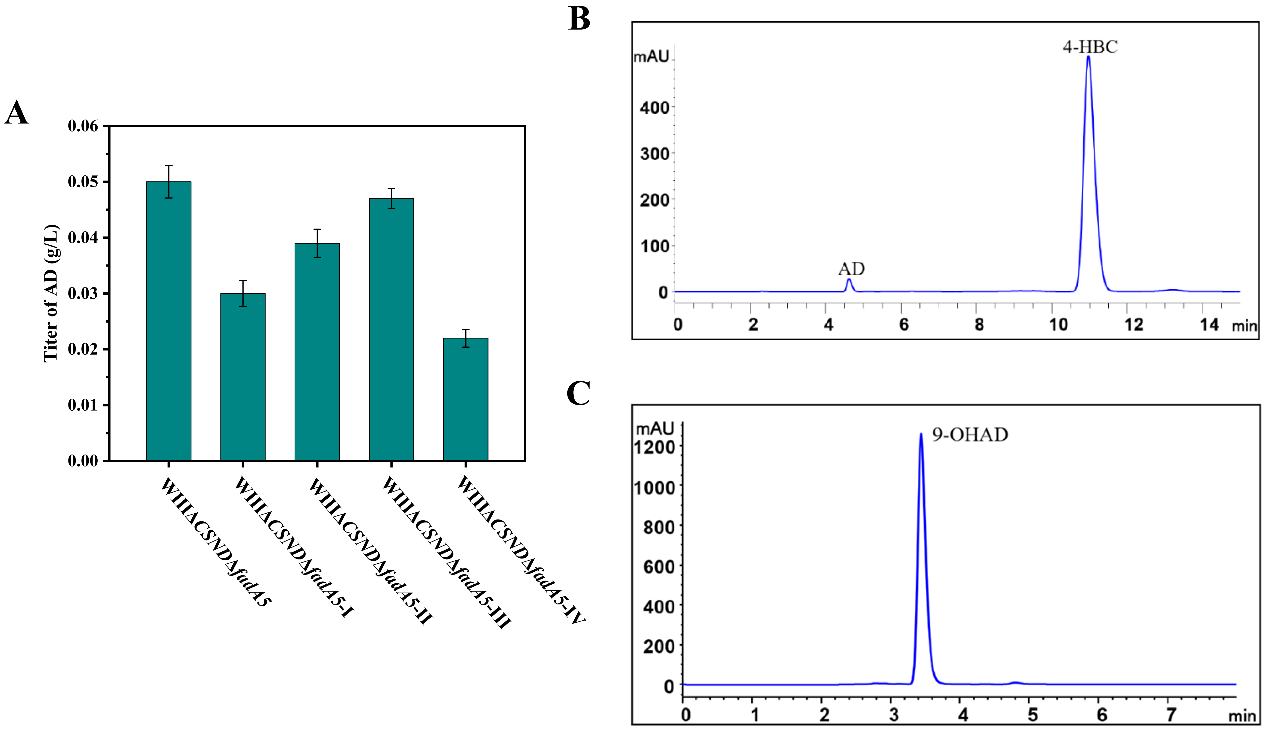


**Figure S6.** Analysis of metabolite profiles derived from sterols conversion by 4-HBC and 9-OHAD-producing strains. (A) Effects of the individual or combined over-expression of *atf1*, *chsH4* and *chsE6* on AD production in 4-HBC-producing strains. (B) HPLC profile of metabolites derived from conversion of 30 g/L phytosterols by strains WIIIΔ*CSND*Δ*fadA5*-IV in a 5-L bioreactor. (B) HPLC profile of metabolites derived from conversion of 30 g/L phytosterols by strain NwIB-IΔ*CSND*-V in a 5-L bioreactor. All assays were performed in triplicate with three independent measurements. Standard deviations of the biological replicates are represented by error bars.

**Table S1.** Transcriptional changes of the genes flanking *atf1.*

| **Gene No.** | **Multiplier change** | **Description** |
| --- | --- | --- |
| *Mn_4950* | 1.4 | Amino acid ABC transporter permease |
| *Mn_4951* | 1.8 | Glutamate ABC transporter substrate-binding protein |
| *Mn_4952* | 1.0 | Amino acid ABC transporter ATP-binding protein |
| *Mn_4953* | 1.1 | tRNA -methyltransferase MiaB |
| *Mn_4954* | 0.9 | Multispecies: hypothetical protein |
| *Mn_4955* | 1.4 | DUF349 domain-containing protein |
| *Mn_4956* | 2.0 | Hypothetical protein |
| *Mn_4957* | 1.0 | Hypothetical protein |
| *Mn_4958* | 1.6 | tRNA (adenosine(37)-N6)-di-methyltransferase MiaA |
| *Mn_4959* | 0.8 | Diaminopimelate epimerase |
| *Mn_4960* | 0.8 | GTPase HflX |
| *Mn_4961* | 1.0 | PE-PPE domain-containing protein |
| *Mn_4962* (*atf1*) | 46.0 | Thiolase family protein |
| *Mn_4963* (*chsH4*) | 13.1 | Acyl dehydratase |
| *Mn_4964* (*chsE6*) | 8.1 | Acyl-CoA dehydrogenase |
| *Mn_4965* (*kstR3*) | 1.4 | TetR/AcrR family transcriptional regulator |
| *Mn_4966* | 2.0 | Molybdopterin oxidoreductase |
| *Mn_4967* | 0.7 | Acyl-CoA dehydrogenase |
| *Mn_4968* | 1.4 | Hypothetical protein |
| *Mn_4969* | 0.3 | Transcriptional repressor LexA |
| *Mn_4970* | 0.4 | Organic hydroperoxide resistance protein |
| *Mn_4971* | 1.6 | MarR family transcriptional regulator |
| *Mn_4972* | 1.1 | LysM peptidoglycan-binding domain-containing protein |
| *Mn_4973* | 0.6 | Ranscriptional repressor NrdR |
| *Mn_4974* | 0.6 | PhzF family phenazine biosynthesis protein |

**Table S2.** Differential expression of genes in strains WIII and WIIIΔ*kstR3*.

| **Protein accession** | **Protein description** | **Log_2_ FC** |
| --- | --- | --- |
| WP_081843428.1 | PPE family protein | 2.25 |
| Atf1 | transporter | 4.40 |
| ChsH4 | acyl dehydratase | 4.61 |
| ChsH6 | acyl-CoA dehydrogenase | 4.29 |
| CDQ43626.1 | putative transcriptional regulator | -2.04 |
| CDQ46370.1 | MerR family transcriptional regulator | -2.56 |
| CDQ44450.1 | Dipeptidyl aminopeptidase/acylaminoacyl peptidase | -2.86 |

Note: Transcriptome data for WIII and WIIIΔ*kstR3* using 0.5 g/ L phytosterol induced for 72 h. Log_2_ FC indicates the fold changes at transcriptomic level (T) for WIIIΔ*kstR3* compared to strain WIII. The value of |Log2FC| ≥ 2 is used to judge the significance of differential expressed genes.

**Table S3.** Plasmids and primers used in this study.

| **Name** | **Sequence (5’-3’)** | **Source** |
| --- | --- | --- |
| **Plasmids** |  |  |
| **For gene knock-out** |  |  |
| p2NIL | Nonreplicating plasmid for allelic recombination in *Mycolicibacteria*, *Kan^R^* | Dr. T. Parish |
| pGOAL19 | *LacZ*, *Hyg* and *SacB* marker genes cassette-containing vector, *Hyg^R^* | Dr. T. Parish |
| pKO-*fadA5* | p2NIL carrying homologous arms of *fadA5* and the selection markers from pGOAL19 | This work |
| pKO-*ltp2* | p2NIL carrying homologous arms of *ltp2* and the selection markers from pGOAL19 | This work |
| pKO-*ltp3* | p2NIL carrying homologous arms of *ltp3* and the selection markers from pGOAL19 | This work |
| pKO-*ltp4* | p2NIL carrying homologous arms of *ltp4* and the selection markers from pGOAL19 | This work |
| pKO-*atf2* | p2NIL carrying homologous arms of *atf2* and the selection markers from pGOAL19 | This work |
| pKO-*atf1* | p2NIL carrying homologous arms of *atf1* and the selection markers from pGOAL19 | This work |
| pKO-*kstR3* | p2NIL carrying homologous arms of *kstR3* and the selection markers from pGOAL19 | This work |
| pKO-*chsH4* | p2NIL carrying homologous arms of *chsH4* and the selection markers from pGOAL19 | This work |
| pKO-*chsE6* | p2NIL carrying homologous arms of *chsH6* and the selection markers from pGOAL19 | This work |
| pKO-*oppcR* | p2NIL carrying homologous arms of *oppcR* and the selection markers from pGOAL19 | This work |
| pKO-*CSND* | p2NIL carrying homologous arms of *CSND* gene cluster and the selection markers from pGOAL19 | This work |
| pKO-*fadA5* | p2NIL carrying homologous arms of *fadA5* and the selection markers from pGOAL19 | This work |
| **For gene overexpressing** |  |  |
| pMV261 | Shuttle vector of *E. coli*-*Mycolicibacteria*, carrying the heat shock (*hsp60*) promoter, *Kan^R^* | Dr. W. R. Jacobs Jr. |
| p261-*atf1* | pMV261 with *hsp60* controlled *atf1*, *Kan^R^* | This work |
| p261-*sal1* | pMV261 with *hsp60* controlled *sal1*, *Kan^R^* | This work |
| p261-*shy* | pMV261 with *hsp60* controlled *shy*, *Kan^R^* | This work |
| p261-*sal1*&*shy* | pMV261 with *hsp60* controlled *sal1* and *shy*, *Kan^R^* | This work |
| p261-*chsH4* | pMV261 with *hsp60* controlled *chsH4*, *Kan^R^* | This work |
| p261-*chsE6* | pMV261 with *hsp60* controlled *chsE6*, *Kan^R^* | This work |
| p261-*kstR3* | pMV261 with *hsp60* controlled *kstR3*, *Kan^R^* | This work |
| p261-*atf1*&*chsH4* | pMV261 with *hsp60* controlled *atf1* and *chsH4*, *Kan^R^* | This work |
| **For gene supplementation** |  |  |
| pMV306 | Integration vector in *Mycolicibacteria* species, *Kan^R^* | Dr. W. R. Jacobs Jr. |
| p306-*atf1* | pMV306-*hsp60*-*atf1*, integrative into *Mycolicibacteria* genome DNA, *Kan^R^* | This work |
| p306-*sal1* | pMV306-*hsp60*-*sal1*, integrative into *Mycolicibacteria* genome DNA, *Kan^R^* | This work |
| p306-*shy* | pMV306-*hsp60*-*shy*, integrative into *Mycolicibacteria* genome DNA, *Kan^R^* | This work |
| p306-*sal1*&*shy* | pMV306-*hsp60*-*atf1* and *shy*, integrative into *Mycolicibacteria* genome DNA, *Kan^R^* | This work |
| p306-*chsH4* | pMV306-*hsp60*-*chsH4*, integrative into *Mycolicibacteria* genome DNA, *Kan^R^* | This work |
| **Primers** |  |  |
| **Gene deletion** |  |  |
| *fadA5*-UF | CCGaagcttGTTCCTTCTTGTAGAGCTCCCACTG | This work |
| *fadA5*-UR | AATTgaattcGTACTGGGTGACGCAGCCGCCGATG | This work |
| *fadA5*-DF | CGCgaattcGACATGGACAAGGTCAACGTCAACG | This work |
| *fadA5*-DR | TTAAgcggccgcGGTCGCAGATCAGGATCGGGATCT | This work |
| *ltp2*-UF | AATTctgcagACCAGGCTCGCCATCAACGGGGTC | This work |
| *ltp2*-UR | GACTaagcttATCCACGGCATGAACGGGATCGCC | This work |
| *ltp2*-DF | GCGaagcttGTCCATCGTGAAGGTGACCAGACC | This work |
| *ltp2*-DR | TTAAgcggccgcGAGATGGACTGGCGCATCATGAAG | This work |
| *ltp3*-UF | GCGctgcagGAACATCAGCACGGTGATGTCCTTG | This work |
| *ltp3*-UR | AATTaagcttGGTGAGGGCTGGAAGCTCACCGAGGC | This work |
| *ltp3*-DF | GTCAaagcttCGGAATCAGCCAGCGCACGGTCGAT | This work |
| *ltp3*-DR | AATTggatccGGATTACCTTGCCGGTCGCGCCTTC | This work |
| *ltp4*-UF | TAATctgcagGCCTCGGTGAGCTTCCAGCCCTCAC | This work |
| *ltp4*-UR | TAATaagcttGTCAATCCGTCCGGCGGCACCCTGG | This work |
| *ltp4*-DF | ATATaagcttGAAGGCGCGACCGGCAAGGTAATCC | This work |
| *ltp4*-DR | CGCGggtaccTTCTTAGAACCTGTTCTACTCGATG | This work |
| *atf1*-UF | TAATaagcttCTGGTTGGGGGCCAGGTAGGTGCCG | This work |
| *atf1*-UR | TAATggtaccGACCTGCTGCACCGACAAGCCGGCA | This work |
| *atf1*-DF | ATTAggtaccGCCGGTGGTATGCACCATGTCTGCG | This work |
| *atf1*-DR | ATTAggatccGATCACGTGTTCGGCGGCGGCGATA | This work |
| *atf2*-UF | AATTggatccGTTGATGCGCGATGCGCCGTTC | This work |
| *atf2*-UR | AATTgaattcGCCGTAATGCGATTCGGCGATG | This work |
| *atf2*-DF | AATTgaattcTACTCCGGTAGCGGTTGCAGCC | This work |
| *atf2*-DR | AATTggtaccACCGGCAGTATCGGGCCTTGAG | This work |
| *kstR3*-UF | AATTggatccAGTCGTGGCGCCGTATCTGCC | This work |
| *kstR3*-UR | TAATaagcttGACATCGCGCATCTGGATCTGCTCG | This work |
| *kstR3*-DF | TAATaagcttTCCATCCTGCGCGACCTGCCG | This work |
| *kstR3*-DR | AATTggtaccCTCCACCACCGCCTATGAGCGTG | This work |
| *chsH4*-UF | ATATaagcttCGCGGAATTCACGATCGAACAATGG | This work |
| *chsH4*-UR | AATTggtaccTCAGTCCCCCTCCAGGACGAGAG | This work |
| *chsH4*-DF | AATTggtaccATCGGTATGGCGATGACACCGG | This work |
| *chsH4*-DR | AATTggatccGCCAGTTCGATGCCGAAATCCCT | This work |
| *chsE6*-UF | TAATaagcttAATGCCGAACTGGCCCGCACAAC | This work |
| *chsE6*-UR | AATTgaattcCTCGGGTAACTGGACAGCATGCAGAGC | This work |
| *chsE6*-DF | AATTgaattcGTGACCGCCGATGCGTTGGCC | This work |
| *chsE6*-DR | AATTggatccGTGGTGCGGGTGTTCAACGATCGCG | This work |
| *opccR*-UF | TTAAagcttgTCGCTTCGCGGCAACGGACCA | This work |
| *opccR*-UR | GCCcatatgGTCAAGCGCGCGGTCCGAGC | This work |
| *opccR*-DF | GACcatatgGCCGGTAACGACATAATGCATGTCGG | This work |
| *opccR*-DR | ATAggatccGCAGCAATCGTTCTCGCGACCG | This work |
| *CSND*-UF | AATTaagcttCGACTGCGCTCCGCCTCGGCC | This work |
| CSND-UR | AATTggtaccGGGCAACGACCTTGTGCATGGTCGCC | This work |
| *CSND*-DF | AATTggtaccCAGCCACCTGGCGGCACTGGACATG | This work |
| *CSND*-DR | AATTggatccGCGGCGACGGCGAACGGTTGGAAC | This work |
| **Gene augmentation** |  |  |
| *atf1*-F | TAGCggatccATTGGGTTTGCGTGGTGACGCAGC | This work |
| *atf1*-R | TAGCaagcttTCAGTCCCCCTCCAGGACGAGAGCT | This work |
| *chsH4*-F | AATTgaattcATGACCGACCCGATGGTGCG | This work |
| *chsH4*-R | TTGGaagcttCTATTCGGCCGCGGTGTAATGCAAG | This work |
| *chsE6*-F | AATTggatccAATGGCGCTGGCCCTCACCGA | This work |
| *chsE6*-R | TATAaagcttTCATGCGCGCGGTAGCCCGA | This work |
| *kstR3*-F | AATTgaattcATGACCGCGGCCGACACCGAAC | This work |
| *kstR3*-R | ATATaagcttTCAGTCCCGGCCCGCCACCG | This work |
| *atf1&chsH4*-F | AATTggatcctTTGGGTTTGCGTGGTGACGCAGC | This work |
| *atf1&chsH4*-R | TTGGaagcttCTATTCGGCCGCGGTGTAATGCAAG | This work |

Notes: The restriction enzyme sites are indicated with lowercase letters.
